# Integrative Analysis of Biomarkers Through Machine Learning Identifies Stemness Features in Colorectal Cancer

**DOI:** 10.3389/fcell.2021.724860

**Published:** 2021-09-08

**Authors:** Ran Wei, Jichuan Quan, Shuofeng Li, Hengchang Liu, Xu Guan, Zheng Jiang, Xishan Wang

**Affiliations:** Department of Colorectal Surgery, National Cancer Center/National Clinical Research Center for Cancer/Cancer Hospital, Chinese Academy of Medical Sciences and Peking Union Medical College, Beijing, China

**Keywords:** tumor immune microenvironment, colorectal cancer, cancer stem cell, N6-methyladenosine, machine learning

## Abstract

**Background:** Cancer stem cells (CSCs), which are characterized by self-renewal and plasticity, are highly correlated with tumor metastasis and drug resistance. To fully understand the role of CSCs in colorectal cancer (CRC), we evaluated the stemness traits and prognostic value of stemness-related genes in CRC.

**Methods:** In this study, the data from 616 CRC patients from The Cancer Genome Atlas (TCGA) were assessed and subtyped based on the mRNA expression-based stemness index (mRNAsi). The correlations of cancer stemness with the immune microenvironment, tumor mutational burden (TMB), and N6-methyladenosine (m6A) RNA methylation regulators were analyzed. Weighted gene co-expression network analysis (WGCNA) was performed to identify the crucial stemness-related genes and modules. Furthermore, a prognostic expression signature was constructed using the Lasso-penalized Cox regression analysis. The signature was validated *via* multiplex immunofluorescence staining of tissue samples in an independent cohort of 48 CRC patients.

**Results:** This study suggests that high-mRNAsi scores are associated with poor overall survival in stage IV CRC patients. Moreover, the levels of TMB and m6A RNA methylation regulators were positively correlated with mRNAsi scores, and low-mRNAsi scores were characterized by increased immune activity in CRC. The analysis identified 34 key genes as candidate prognosis biomarkers. Finally, a three-gene prognostic signature (PARPBP, KNSTRN, and KIF2C) was explored together with specific clinical features to construct a nomogram, which was successfully validated in an external cohort.

**Conclusion:** There is a unique correlation between CSCs and the prognosis of CRC patients, and the novel biomarkers related to cell stemness could accurately predict the clinical outcomes of these patients.

## Introduction

Colorectal cancer is among the most common and lethal cancers of the digestive system (Sanoff et al., 2007). Although neoadjuvant chemoradiotherapy and immunotherapy offer good prospects for operable colorectal cancer cases, the 5-year survival rates remain low in cases with advanced disease (Bray et al., 2018). Following advances in individualized tumor treatment, a remarkable tumor heterogeneity was discovered and shown to be closely associated with chemoresistance, radiosensitivity, and patient survival (Chen et al., 2016). Cancer stem cell (CSC) traits, a crucial part of cancer heterogeneity, are considered to be crucial drivers of the prognosis and response to therapy (Park et al., 2012; Chen et al., 2016).

Mounting evidence suggests the existence of CSCs in colorectal cancer, with studies revealing their roles in metastasis, drug resistance, and continual adaptation of cancer cells to the changing microenvironment (Zeuner et al., 2014; Lenos et al., 2018). It has been demonstrated that accumulated epigenetic and genetic variability allows CSC to evolve and thereby continue tumor growth and maintenance, which is closely associated with the alteration of the tumor microenvironment (TME) (Wicha et al., 2006; Kreso and Dick, 2014). Recent studies demonstrated that colon cancer cells with stem-cell-like properties can promote tumor development, which is regulated by the TME and not a fully autonomous behavior of individual cells (Ricci-Vitiani et al., 2007; Choi et al., 2009). Cancer stemness encompasses both the stemness phenotype of bona fide CSCs and the intrinsic potential for differentiation into colon cancer cells, which is considered a fundamental underlying property of malignancy (Michieli et al., 2004; Cheng et al., 2006). However, most related theories have not been confirmed *in vivo* or translated into clinical research, which can be attributed to the integrated and complex cancer ecosystem.

The interaction between the immune system and CSCs is still controversial, as the increased tumorigenicity of CSCs reveals that they can promote oncogenic immunomodulation in colon cancer (Condeelis and Pollard, 2006; Baharom et al., 2020). Moreover, cancer cells and CSCs with high stemness exhibit decreased expression of major histocompatibility complex (MHC) molecules, thereby promoting immune evasion and decreasing the activity of antitumor immune cells (Sultan et al., 2017). However, an integrated understanding of colorectal cancer stemness, including its interactions with the tumor immune microenvironment, still requires further research. To evaluate the role of stemness in tumor pathogenesis and the vital factors leading to dedifferentiation and acquisition of stem-cell-like properties in colorectal cancer, artificial intelligence and bioinformatic methods could be employed to further understand cancer stemness (Sokolov et al., 2016; Malta et al., 2018). One-class logistic regression (OCLR) can be used to extract epigenetic and transcriptomic features from normal stem cells and their differentiated progeny, including induced pluripotent stem cells and embryonic stem cells with different level of stemness. Notably, this approach could also be used to identify the stem cell signatures and quantify cancer stemness *via* a multi-omics analysis.

In this study, we hypothesized that cancer stemness may confer immunosuppressive properties on tumors and mediate the prognosis. To verify it, we developed the immune score construct and identified the proportions of immune cells using vector regression. We then assessed of correlations the stemness indices with molecular features and identified a stemness-related gene signature. The prognostic signature was explored together with specific clinical features to construct a nomogram, which was successfully validated in an external cohort, which might be helpful in evaluating the prognosis of colon and rectal cancer patients.

## Materials and Methods

### Data Sources

Within The Cancer Genome Atlas (TCGA) database,^1^ we identified the transcriptome profiling by RNA-sequencing (RNA-seq), single nucleotide variants (SNV), and the corresponding prognostic and clinicopathological information of the colon and rectal cancer set. Moreover, the Ensemble IDs were converted to gene symbols based on the Ensemble database.^2^ The RNA-seq results of 433 tissues and 408 colon cancer samples were obtained from TCGA-colon adenocarcinoma (COAD), and 221 tissues and 208 rectal cancer samples were obtained from TCGA-rectum adenocarcinoma (READ) with the fragments per kilobase of transcript million mapped reads (FPKM) method (Zeng et al., 2018). The SNV results were obtained from COAD and READ with the VarScan2 Variant Aggregation and Masking.

### Colorectal Cancer Patients

Colorectal cancer specimens that underwent surgical resection from January 2006 through December 2012 were approved by the Pathology Department of the Cancer Institute and Hospital, Chinese Academy of Medical Sciences. All of the patients provided informed consent. Patients who met the following criteria were included: a) did not undergo neoadjuvant radiotherapy or chemotherapy before the surgery and b) with complete clinicopathological information, including sex, age, tumor location, TNM classification, and follow-up time. Moreover, the patients in this study were followed up every 3 months until January 1, 2018. Forty-eight tumor tissues and adjacent normal tissues were collected from colorectal cancer patients with untreated stage III to stage IV. This study has been approved by the Ethics Committee of Cancer Hospital, Chinese Academy of Medical Sciences.

### mRNAsi in Subtypes

Unsupervised learning, as a machine learning technique, was employed in the field of medical data mining, and Malta et al. (2018) drew two stemness indices based on reflected epigenetic regulation features and transcriptome, respectively, named mRNAsi and mDNAsi with OCLR. The mRNAsi is used to identify cancer stemness features and assess the degree associated with oncogenic dedifferentiation, which could be a quantitative form of cancer CSCs. Higher mRNAsi scores are related with cancer biological processes in CSCs and more tumor dedifferentiation based on the histopathological grades. We divided colon and rectal cancer patients into high-mRNAsi group and low-mRNAsi group based on the cutoff value determined by the median mRNAsi index.

### Differentially Expressed Genes

The “limma” packages in R language were employed to identify differentially expressed genes in the expression data from TCGA, and the Wilcoxon test was used in the analysis processing (Yoshihara et al., 2013). The | log2-fold change| > 1 and false discovery rate (FDR) < 0.05 were considered to be the cutoff criteria for the selection of differentially expressed genes (DEGs) between colon and rectal cancer and normal sets. The volcano plot and heatmap were drawn with the “pheatmap” package in R.

### Estimation of the Immune Microenvironment and Infiltrating Immune Cells

We employed the Estimation of Stromal and Immune cells in Malignant Tumors using Expression data (ESTIMATE) to evaluate the immune score and immune cell infiltration level, for each colon and rectal cancer sample from TCGA (Song et al., 2017). Then, we evaluated the enrichment levels of the 29 immune signatures in each colon and rectal cancer specimen by the single-sample gene set enrichment analysis (ssGSEA) score (Ritchie et al., 2015). The colon and rectal cases could be classified into three subgroups based on the ssGSEA score and hierarchical clustering. Besides, we evaluated the 22 human immune cell subsets of every colon and rectal cancer sample with Cell type Identification by Estimating Relative Subsets of RNA Transcripts (CIBERSORT) web portal^3^ and 1,000 permutations (Barbie et al., 2009). The CIBERSORT deconvolution algorithm output had a *p*-value < 0.05 and was considered accurate and successful deconvolution. Moreover, the output estimates would be normalized for each sample to add up to one, enabling their direct interpretation as cell fractions for comparison across different groups. The package “Genefilter” R was employed to identify each sample.

### Weighted Gene Co-expression Network Analysis

We used the weighted gene co-expression network analysis (“WGCNA”) R package to establish the co-expression network of differentially expressed genes (Chai et al., 2019). We employed Pearson’s correlation matrices, co-expression similarity matrix, and average linkage method to evaluate the correlations among the included genes. We used the function Amn = | Cmn| β (Cmn = Pearson’s correlation between gene-m and gene-n; Amn = adjacency between gene m and gene n; β representing soft thresholding parameter) to distinguish the strength of correlations and build a weighted adjacency matrix with a scale-free co-expression network. We used a topological overlap matrix (TOM) to evaluate the connectivity and dissimilarity of the co-expression network established with an appropriate β value. Based on the TOM dissimilarity measurements, the average hierarchical linkage clustering could be established, and we set the minimum genome to 30 to build module dendrograms.

In order to confirm the key modules and genes, we set the module membership (MM) and gene significance (GS) to be the measure used to identify the correlation between genes and mRNAsi and epigenetically regulated mRNAsi (EREG-mRNAsi). The module eigengenes (MEs) were defined as the significant components of principal component analysis (PCA) for each gene module, where the expression level of every gene could be grouped with a distinct feature. We used a log10 transformation of the *p*-value (GS = lgp) for the linear regression of correlations between clinical phenotypes and gene expression. We used module significance (MS) to represent the correlation between clinical traits and gene expression calculated using the average GS in the module. We used a cutoff of < 0.25 to merge highly similar modules, which could help cluster the key genes. The thresholds for the screening of key module genes were set as cor. gene GS > 0.5 and cor. gene MM > 0.6.

### Gene Set, Ontology, and Pathway Enrichment Analysis

The package “clusterProfiler” in R was employed to evaluate the enrichment analysis of Kyoto Encyclopedia of Genes and Genomes (KEGG) and Gene Ontology (GO) to reveal the key biological functions of the module genes (Yang et al., 2018). We set *p*-value < 0.05 and an FDR < 0.05 as the statistically significant criteria to output. The whole transcriptome of all tumor samples was employed for GSEA, and only gene sets with NOM *p* < 0.05 and FDR *q* < 0.05 were set as statistically significant criteria.

### Multiplex Immunofluorescence Staining

To evaluate the expression and distribution of three key genes related to tumor stemness in colorectal cancer and normal tissues, we performed multiplex immunofluorescence staining using the PANO 7-plex IHC kit (Cat. No. 0004100100; Panovue, Beijing, China) and Tyramide Signal Amplification Fluorescence Kit (Panovue, Beijing, China) (Yu et al., 2012). We established the colorectal cancer tissue microarrays (TMAs) consisting of primary tumor, metastatic tumor, and matched normal tissue from cancer patients who had been confirmed by pathological examination with hematoxylin and eosin (H&E) staining. Each of these tissues was cut into pieces of 1.0 mm and attached to the slides (5 mm thick) from the TMAs. The TMAs were incubated with anti-PARPBP (ab211634; 1:100; Abcam, Cambridge, United Kingdom), anti-KNSTRN (ab122769; 1:100; Abcam, Cambridge, United Kingdom), and anti-KIF2C (12139-1-AP; 1:200; Proteintech, Rosemont, IL, United States) antibodies at 4°C overnight, and then with horseradish peroxidase-conjugated secondary antibody and tyramide. A microwave was used to heat-regenerate the TMAs after each Tyramide Signal Amplification step. We used 4′,6-diamidino-2-phenylindole (DAPI) to counterstain the cell nuclei. The multiplex immunofluorescence image analysis is shown in Supplementary Materials.

### Statistical Analysis

In this study, the colon and rectal cancer specimens were allocated into training and testing groups randomly through the 2:1 ratio with the package “caret,” in order to promote the generalization ability of the model. Moreover, we established the Lasso-penalized Cox regression model to identify the most significant survival-related gene signatures with the overall survival (OS) of patients. We set 10-fold cross-validation as the criteria to prevent overfitting with the penalty parameter lambda.1se (Goeman, 2010). Then, the time-dependent receiver operating characteristic (ROC) curve and the area under the curve (AUC) were employed to identify the prognostic accuracy of the three-gene signature model in the training and testing groups with the package “survival ROC” (Heagerty et al., 2000). The median of risk scores was set as the cutoff value to the separate patients into high-risk and low-risk score groups. We employed the Kaplan–Meier survival analysis and the log-rank test to evaluate difference in OS between different groups. The final (forward and backward elimination methods) multivariate Cox regression analysis was employed to evaluate the independence of predictors and established three signatures and a nomogram. Then, we validated the performance of the Cox model internally and externally with the bootstrap method. Bootstrap-corrected OS rates were calculated by averaging the Kaplan–Meier estimates based on 2,000 bootstrap samples.

## Results

### Colon and Rectal Cancer Patients Could Be Subtyped Using the mRNAsi

To explore the mRNAsi in colon and rectal cancer, we obtained the transcriptome profiling for the gene expression and clinical information of colon and rectal cases from the TCGA database, and the analysis workflow is shown (Supplementary Figure 1).

**FIGURE 1:**
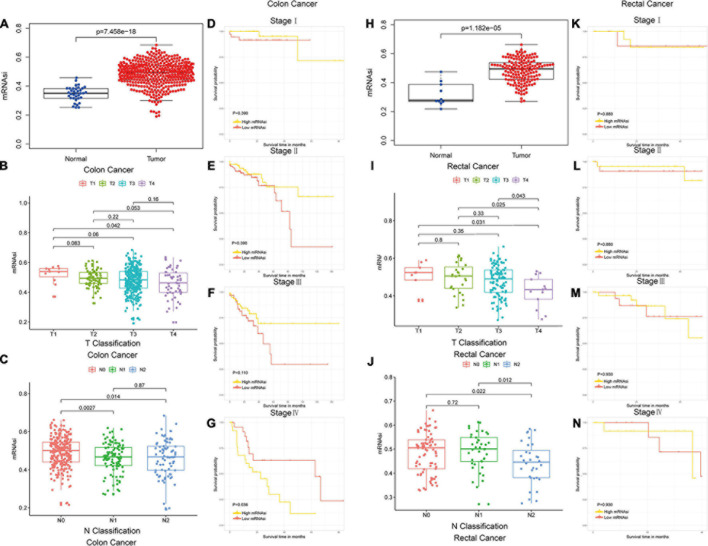
Colon and rectal cancer patients could be subtyped using the mRNAsi. **(A)** Differences in mRNAsi between normal and colon cancer tissues. **(B,C)** Comparison of mRNAsi in different T stages **(B)** or N stages **(C)** in colon cancer. **(D–G)** Kaplan–Meier survival curves of mRNAsi in colon cancer. *p* < 0.05 indicates statistical significance. **(H)** Differences in mRNAsi between normal and rectal cancer tissues. **(I,J)** Comparison of mRNAsi in different T stages **(I)** or N stages **(J)** in rectal cancer. **(K–N)** Kaplan–Meier survival curves of mRNAsi in rectal cancer. *p* < 0.05 indicates statistical significance.

The mRNAsi of colon and rectal cancer tissues was significantly higher than that of normal tissues (Wilcoxon rank sum test, *p* < 0.05, Figures 1A,H), which suggested that the level of stemness is associated with tumor development. The mRNAsi among different stages of colon and rectal cancer did not show significant differences (Mann–Whitney *U*-test, *p* < 0.05, Figures 1B,C,I,J), which suggested the mRNAsi may not be closely related to the clinical stage in many tumor types (Bai et al., 2020; Mao et al., 2021; Pei et al., 2020). In our study, the colon and rectal cancer patients were divided intohigh-mRNAsi and lowmRNAsi groups based on the cutoff value determined by the median mRNAsi index (Figures 1D–G,K–N). Stage IV colon cancer patients with higher mRNAsi had a significantly shorter OS than those with lower mRNAsi (log-rank test, *p* < 0.05, Figure 1G). These results suggested that the mRNAsi of colon and rectal cancer is closely associated with the prognosis of stage IV colon cancer patients.

### Differences in Gene Mutations and the Expression of m6A RNA Methylation Regulators Among the mRNAsi Subtypes

We compared the mutational landscape between colon and rectal cancer patients in the high- and low-mRNAsi groups. A higher proportion of APC (80.7%) and TP53 (61.9%) mutations were found in the high-mRNAsi group than the proportion of APC (70.7%) and TP53 (51.2%) mutations in the low-mRNAsi group of colon cancer patients, and the difference was statistically significant (Supplementary Figures 2, 3).

Moreover, high mRNAsi was significantly associated with increasing expression of m6A RNA methylation regulators in colon and rectal (Wilcoxon rank sum test, ^∗^*p* < 0.05; ^∗∗^*p* < 0.01; ^∗∗∗^*p* < 0.001, Supplementary Figures 4A,B). Correlation analysis was performed in high- and low-mRNAsi subgroups of colon and rectal cancer patients (Supplementary Figures 4C–F). The result suggested that high cancer stemness could be significantly associated with the high expression of m6A RNA methylation regulators and regulated their correlation.

### Differences of Cell Stemness and Immune Microenvironment Between the mRNAsi Subtypes

**FIGURE 2:**
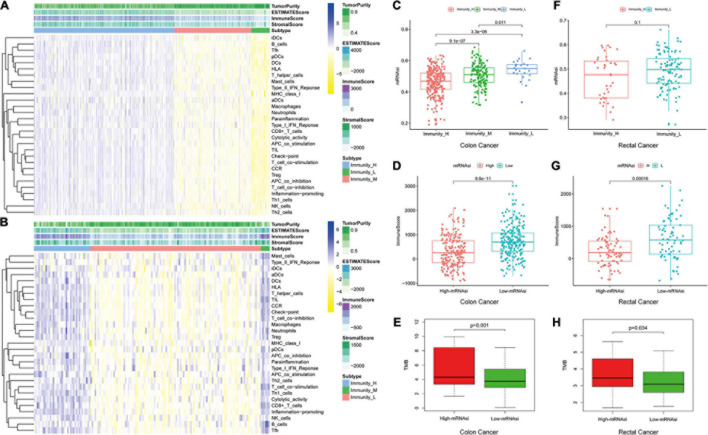
Hierarchical clustering of colon and rectal cancer yields three stable subtypes in four different datasets named Immunity_H, Immunity_M, and Immunity_L. Tumor purity, stromal score, and immune score were evaluated by ESTIMATE. **(A)** The colon cancer patients in the TCGA-COAD database. **(B)** The rectal cancer patients in the TCGA-READ database. **(C,F)** Comparison of the mRNAsi levels between three colorectal cancer subtypes. Mann–Whitney *U*-test. **(D,G)** Comparison of the immune cell infiltration levels between different mRNAsi groups of colorectal cancer. Mann–Whitney *U*-test. **(E,H)** Comparison of the TMB levels between different mRNAsi groups of colorectal cancer. Kruskal–Wallis rank sum test.

We used 29 immune-associated gene sets to quantify the enrichment levels of different immune cell functions, pathways, and types in colon and rectal cancer samples based on ssGSEA scores (Chai et al., 2019). The ssGSEA scores of the 29 gene sets were employed for hierarchical clustering of immune subtypes into high, moderate, and low groups (Figures 2A,B). We also evaluated the immune score using ESTIMATE, and the immune scores were higher in the high subtype than those in the low subtype (Wilcoxon rank sum test, *p* < 0.001) (Ritchie et al., 2015). The mRNAsi in the high subtype was higher than in the low subtype for colon cancer patients. However, there is no significant difference for rectal cancer patients (Kruskal–Wallis test, Wilcoxon rank sum test, *p* < 0.001, Figures 2C,F). Moreover, we found that immune score was lower in the high-mRNAsi group than in the low-mRNAsi group for both colon and rectal cancer (Wilcoxon rank sum test, *p* < 0.001, Figures 2D,G). Moreover, immune score has been proven lower in the high-mRNAsi group than in the low-mRNAsi group for gastric cancer (Mao et al., 2021). In addition, when comparing the tumor mutational burden (TMB) in groups with different levels of stemness index, we observed the opposite trend, with the level of TMB increasing from the low-mRNAsi group to the high-mRNAsi group (low mRNAsi < high mRNAsi) (Wilcoxon rank sum test, *p* < 0.001, Figures 2E,H).

**FIGURE 3:**
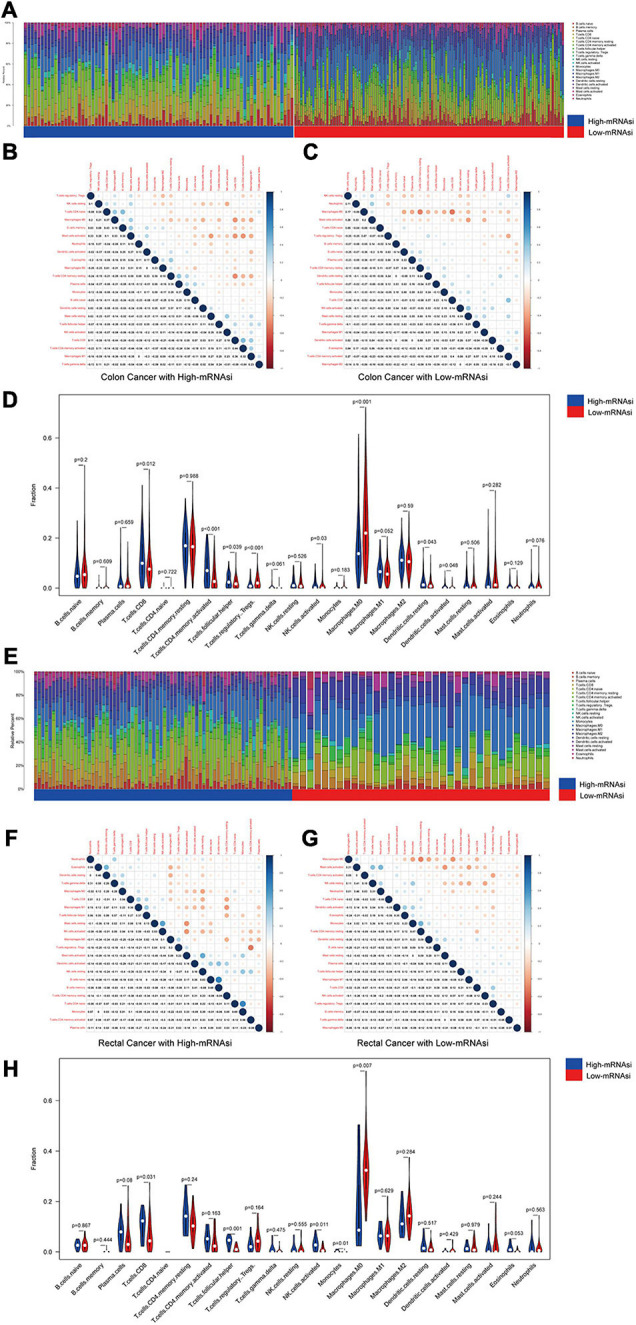
Composition of immune cells in different mRNAsi groups and correlation analysis. **(A)** Barplot showing the fractions of 22 immune cells of colon cancer patients in the TCGA-COAD database. Column names of the plot were the sample ID. **(B)** Heatmap showing the correlation between immune cells of colon cancer cases in the high-mRNAsi group. **(C)** Heatmap showing the correlation between immune cells of colon cancer cases in the low-mRNAsi group. The shade of each tiny color box represented the corresponding correlation value between two cells. **(D)** Comparison of the proportions of immune cell subsets between different mRNAsi groups of colon cancer. ANOVA test, *p*-values are shown. **(E)** Barplot showing the fractions of 22 immune cells of rectal cancer patients in the TCGA-READ database. Column names of the plot were the sample ID. **(F)** Heatmap showing the correlation between immune cells of rectal cancer cases in the high-mRNAsi group. **(G)** Heatmap showing the correlation between immune cells of rectal cancer cases in the low-mRNAsi group. The shade of each tiny color box represented the corresponding correlation value between two cells. **(H)** Comparison of the proportions of immune cell subsets between different mRNAsi groups of rectal cancer. ANOVA test, *p*-values are shown.

We investigated the proportions and differences of tumor-infiltrating immune cell subsets between the high- and low-mRNAsi subgroups of colon and rectal cancer patients using the CIBERSORT algorithm and the LM22 gene signature (Figures 3A,E). To further confirm the relationships among 22 tumor-infiltrating immune cell types, we performed correlation analysis. The results revealed a positive correlation between CD8^+^ T cells and M1 macrophages in the high-mRNAsi subgroup. The CD8^+^ T cells were more strongly negatively correlated with mast cells and resting memory CD4^+^ T cells in the high-mRNAsi group than the low-mRNAsi group of colon cancer patients (Figures 3B,C,F,G). Eight types of tumor-infiltrating immune cells were correlated with the mRNAsi in colon cancer and five types in rectal cancer (Wilcoxon rank sum, *p* < 0.05) (Figures 3D,H). Among them, six types of tumor-infiltrating immune cells were positively correlated with high mRNAsi in colon cancer, namely, CD8^+^ T cells, resting NK cells, activated memory activated CD4^+^ T cells, follicular helper T cells, and resting and activated dendritic cells. Two types of immune cells were positively correlated with low mRNAsi, namely, regulatory T cells and M1 macrophages.

The immune checkpoint molecules are significant for immunotherapy, including MHC classes I and II, and we investigated the potential correlation between the mRNAsi and immune checkpoint molecules. Human leukocyte antigen (HLA) gene expression was enriched in the low-mRNAsi subgroup in colon and rectal cancer (Supplementary Figure 5). Besides, a higher expression of m6A RNA methylation regulators and a lower level of immune checkpoint molecules were found in the high-mRNAsi group than in the low-mRNAsi group. The result suggested that the high level of m6A RNA methylation regulators was associated with a low level of immune checkpoint molecules, which was also proved in pancreatic ductal adenocarcinoma and breast cancer (Zhou et al., 2021).

Therefore, the high-mRNAsi group exhibited higher levels of TMB and a lower immune score, which indicated that tumor stemness may be negatively correlated with tumor immunity and positively correlated with TMB, which is related closely with the production of neoantigens in tumors and associated with response to immune checkpoint inhibitors (Zhou and Li, 2021).

### The Screening of DEGs and the Identification of mRNAsi-Related Modules

**FIGURE 4:**
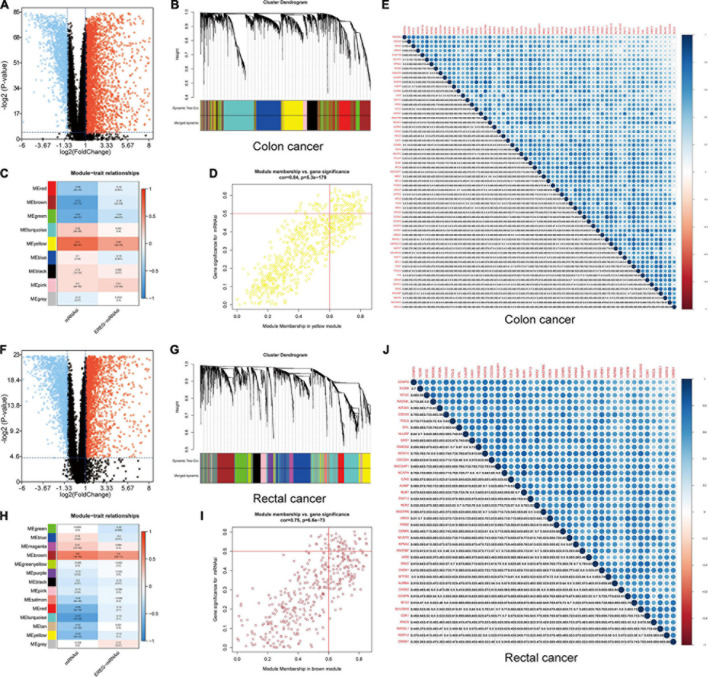
Identification of DEGs and stemness-related key modules in colon and rectal cancer. **(A,F)** Differentially expressed genes: red indicates upregulated genes, blue indicates downregulated genes, and black indicates genes excluded by DEG screening criteria. **(B,C,G,H)** Identification of a co-expression module in colon and rectal cancer. Each piece of the leaves on the cluster dendrogram corresponded to a gene, and those genes with similar expression patterns compose a branch. Correlation between gene modules and mRNAsi scores or EREG-mRNAsi. The upper row in each cell indicates the correlation coefficient ranging from -1 to 1 of the correlation between a certain gene module and mRNAsi or EREG-mRNAsi. The lower row in each cell indicates the *p*-value. **(D,I)** The scatter plot of the top three important gene modules, and those circles located in the upper right indicate the key genes in these modules. **(E,J)** Heatmap showing the correlation between stemness-related genes of colon and rectal cancer cases. The shade of each tiny color box represented the corresponding correlation value between two cells.

We screened DEGs in datasets of colon and rectal cancer tissues and normal tissues. We identified 6,498 DEGs in colon cancer, 4,528 of which were upregulated and 1,970 downregulated (Figure 4A). In rectal cancer, 2,072 DEGs were upregulated and 1,776 were downregulated (Figure 4F).

A gene co-expression network was established to classify the genes into biologically significant modules based on the average linkage hierarchical clustering strongly associated with the mRNAsi in colon and rectal cancer. In this model, we set β = 6 (scale-free *R*^2^ = 0.95) as the soft threshold to construct the scale-free network (Supplementary Figures 6, 7), which yielded 9 gene modules in colon cancer and 14 in rectal cancer (Figures 4B,C,G,H). To further identify the relationship between key gene models and mRNAsi, we set MS as the overall gene expression level of a certain module to identify correlations with stemness phenotypes. We selected the yellow module with a correlation of more than 0.7 in colon cancer and the brown module with a correlation of almost 0.6 in rectal cancer for subsequent analyses (Figures 4B,D,G,I). We set cor. MM > 0.6 and cor. GS > 0.5 as the selection threshold and identified 61 key genes significantly related to mRNAsi in colon cancer, as well as 41 key genes in rectal cancer. The PPI network, GO, and KEGG pathway enrichment analyses were performed to evaluate the protein interactions and principal biological functions of the key genes in colon and rectal cancer (Supplementary Figures 8, 9). By overlapping the mRNAsi-related genes in colon cancer (Figure 4E) and those in rectal cancer (Figure 4J), we obtained 34 overlapped genes and established the PPI network (Supplementary Figures 8, 9). We employed two databases, cBioPortal and Oncomine, to systematically understand the key gene mutational landscape and expression (Supplementary Figures 10, 11; Cerami et al., 2012).

### Prognostic Value of Genes in the mRNAsi-Related Modules in Colon and Rectal Cancer

**FIGURE 5:**
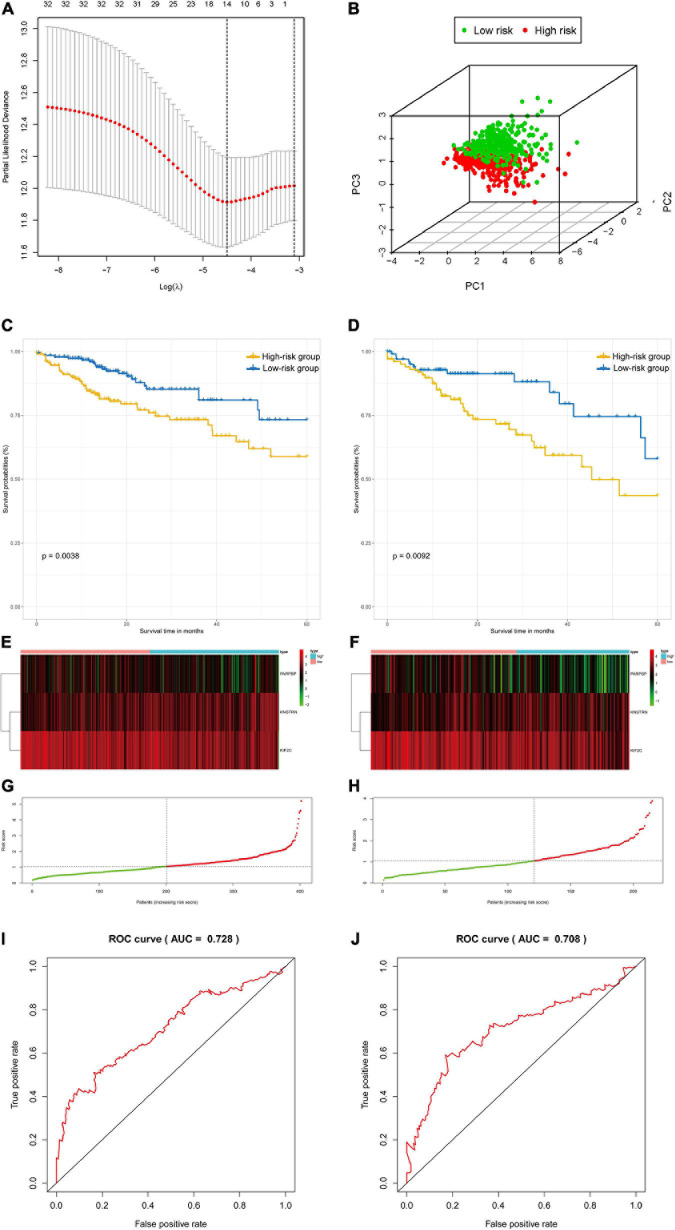
The survival analysis and prognostic performance of the three-gene signature of colon and rectal cancer. **(A)** Lasso of the risk score for colon and rectal cancer between high-risk and low-risk patients in the training group. **(B)** PCA of the risk score for colon and rectal cancer between high-risk and low-risk patients in the training group. **(C)** The Kaplan–Meier test of the risk score for the overall survival of colon and rectal cancer between high-risk and low-risk patients in the training group (log-rank test, *p* < 0.001). **(D)** The prognostic value of the risk score shown by the time-dependent receiver operating characteristic (ROC) curve for predicting the 5-year overall survival training group. **(E)** Risk score curve of the three-gene signature of colon and rectal cancer in the training group. **(F)** Heatmap showed the expression of three genes by risk score of colon and rectal cancer in the training group. **(G)** The Kaplan–Meier test of the risk score for the overall survival of colon and rectal cancer between high-risk and low-risk patients in the testing group (log-rank test, *p* < 0.001). **(H)** The prognostic value of the risk score shown by the time-dependent ROC curve for predicting the 5-year overall survival in the testing group. **(I)** Risk score curve of the three-gene signature of colon and rectal cancer in the testing group. **(J)** Heatmap showed the expression of three genes by risk score of rectal cancer in the testing group.

The colon and rectal cancer patients were divided into training and validation cohorts, and 34 key genes were selected to established a prognostic stemness risk score model using the Lasso algorithm and final (forward and backward elimination methods) multivariate Cox analysis in the training cohort (Figure 5A and Supplementary Figure 12). The poly (ADP-ribose) polymerase 1 binding protein (PARPBP), kinetochore-localized astrin/SPAG5 binding protein (KNSTRN), and kinesin family member 2C (KIF2C) were correlated with a poor prognosis in patients from the training cohort according to the multivariate Cox regression analysis. The risk scores were calculated based on the sum of loci β values and the risk coefficient in the risk prediction model with discrete clinical outcomes with regard to OS (Figure 5B). The prognostic index formula for colon and rectal cancer patients in the training cohorts was as follows: Risk score = [Status of PARPBP ^∗^ (-0.6921)] + [Status of KNSTRN ^∗^ (1.4204)] + [Status of KIF2C ^∗^ (-0.9696)]. This prediction model based on cancer stemness could be a valuable tool for distinguishing among colon and rectal cancer patients. We divided the patients in the training cohorts into a high-risk group and a low-risk group on the basis of the median risk score, which was set as the cutoff value to determine whether the risk of the patient is high or low in the validation cohorts. The association of the risk prediction model and prognosis is shown in Figure 5. The results of survival analysis proved that the OS of the high-risk group was significantly lower than that of the low-risk group according to the Kaplan–Meier curves of the training cohorts (log-rank test, *p* < 0.01, Figure 5C), as well as the Kaplan–Meier curves of the validation cohorts (log-rank test, *p* < 0.01, Figure 5D). The heatmaps of three significant genes and the risk scores for each sample in the training and validation cohorts are shown in Figures 5E–H. Then, ROC curves were employed to verify the validity of the stemness gene-based prediction model in the training and validation cohorts (Figures 5I,J and Supplementary Figure 13). The AUCs were equal to 0.769 at 1 year, 0.683 at 3 years, and 0.728 at 5 years in the training group (Figure 5I and Supplementary Figure 13). Similarly, the AUCs were equal to 0.685 at 1 year, 0.656 at 3 years, and 0.708 at 5 years in the validation group (Figure 5J and Supplementary Figure 13), which showed that the model could achieve satisfactory predictive accuracy in both the training and validation cohorts.

### Validation of the Three-Gene Signature in Stage IV Colorectal Cancer

**FIGURE 6:**
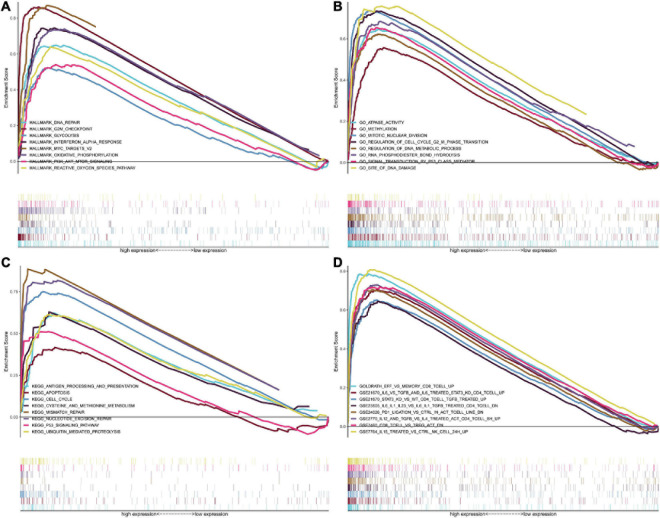
GSEA for samples with high risk and low risk based on the prognostic index of the three-gene signature. **(A)** The enriched gene sets in GO collection by the high-risk sample. Each line representing one particular gene set with a unique color, and the upregulated genes located in the left approaching the origin of the coordinates, by contrast the downregulated lay on the right of the *x*-axis. Only gene sets with NOM *p* < 0.05 and FDR *q* < 0.05 were considered significant, and only several leading gene sets were displayed in the plot. **(B)** The enriched gene sets in KEGG by samples with the high-risk sample, and only several leading gene sets were displayed in the plot. **(C)** Enriched gene sets in HALLMARK collection by samples of the high-risk sample. Only several leading gene sets are shown in the plot. **(D)** Enriched gene sets in C7 collection, the immunologic gene sets, by samples of the high-risk sample. Only several leading gene sets are shown in the plot.

GSEA was conducted to analyze potential biological characteristics of the three-signature genes in colon and rectal cancer patients. As shown in Figure 6A, according to HALLMARK collection defined by MSigDB, the genes in the high-risk group were mainly enriched in cancer stemness-related pathways, such as DNA repair and PI3K–Akt–mTOR signaling. According to GO collection defined by MSigDB, the genes were enriched in functions related to tumor development such as methylation, mitotic nuclear division, signal transduction by P53 class mediators, and DNA damage (Figure 6B). According to KEGG collection defined by MSigDB, the genes were enriched in apoptosis and P53 signaling pathway (Figure 6C). According to the immunological gene set collection defined by MSigDB, multiple immune-function gene sets were enriched in the high-risk group (Figure 6D). To further explore the relationship between the expression of the three-signature genes and the tumor immune microenvironment, we analyzed the corrections between the three genes and 22 types of immune cell infiltration profiles (Supplementary Figure 14).

### Establishment and Validation of the Nomogram

A Cox regression model was applied to the training cohort to identify the predictors of OS. Univariate analyses indicated that age, stage-T, stage-N, stage-M, and cancer stemness risk score group were associated with OS in colorectal cancer patients (*p* < 0.1 in all cases, Table 1). Next, the final (forward and backward elimination methods) multivariate Cox analyses found that age, stage-T, stage-M, and cancer stemness risk score group were independent risk factors for OS (Table 1).

**TABLE 1:** Univariable and multivariable Cox regression analysis of OS in cRC patients.

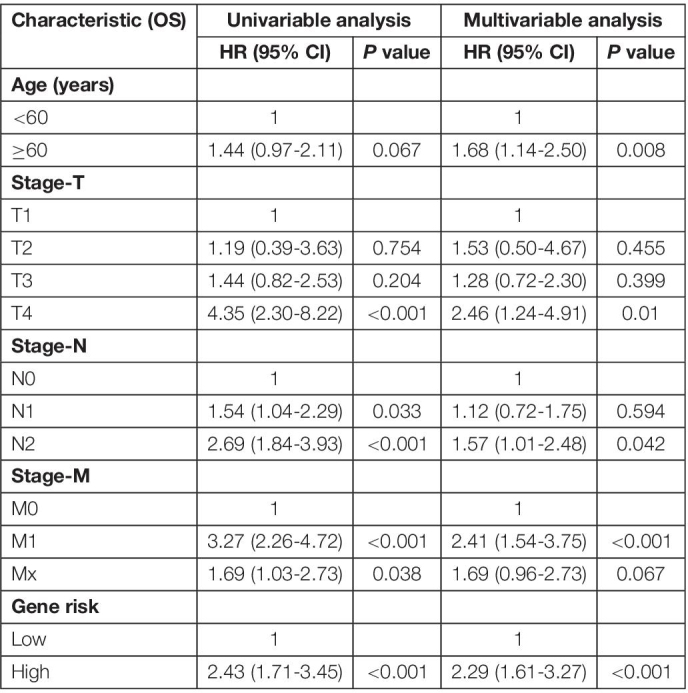

*Multivariate Cox regression analysis are used to calculate the hazard ratios (HRs) and 95% confidence intervals (CIs) for overall survival (OS) in colorectal cancer (CRC) patients. Covariables that are significant in univariable competing risk regression analysis (p < 0.1) are included in the multivariable analysis.*

*Abbreviations: HR, hazard ratio; CI, confidence interval; CRC, colorectal cancer.*

**FIGURE 7:**
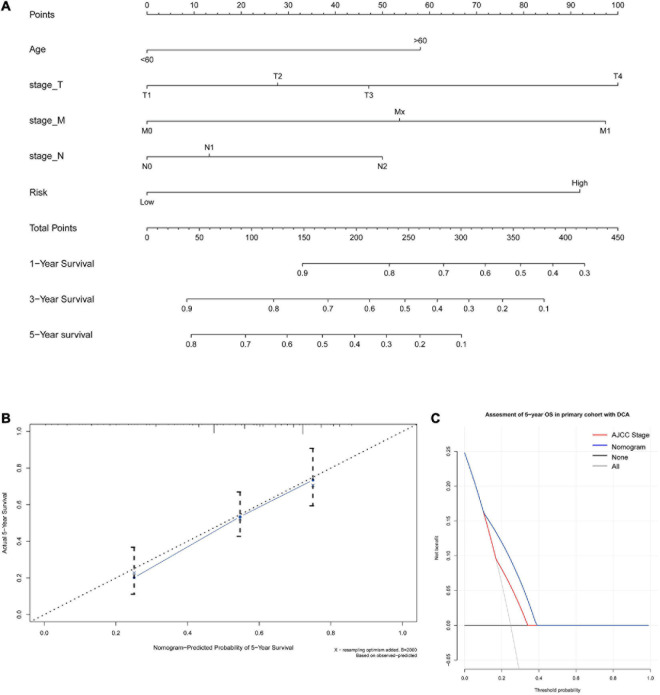
**(A)** A nomogram for predicting 1-, 3-, and 5-year OS in colon cancer. To calculate probability of OS, first determine the value for each factor by drawing a vertical line from that factor to the points scale. “Points” is a scoring scale for each factor, and “total points” is a scale for total score. Then, sum all of the individual values and draw a vertical line from the total points scale to the 1-, 3-, and 5-year OS probability lines to obtain OS estimates. **(B)** Calibration curves for the probability of OS at 5 years. The nomogram cohort was divided into three equal groups for validation. The gray line represents the perfect match between the actual (*y*-axis) and nomogram-predicted (*x*-axis) survival probabilities. Black circles represent nomogram-predicted probabilities for each group, and X’s represent the bootstrap-corrected estimates. Error bars represent the 95% CIs of these estimates. A closer distance between two curves suggests higher accuracy. **(C)** The DCA of nomogram in the training set for 5-year OS.

A nomogram for predicting the 1-, 3-, and 5-year OS was established using these independent variables (Figure 7A). Because age, stage-T, stage-M, and cancer stemness risk score group were predictive for OS in multivariate analysis, these variables were further included in the nomogram. A weighted total score was calculated from these factors, which was applied to predict the 1-, 3-, and 5-year OS of colorectal cancer patients.

The nomogram for predicting the 1-, 3-, and 5-year OS of colorectal cancer patients was developed based on the multivariate model. The model showed good accuracy for predicting the OS, and internal validation was performed using the training cohort with a C-index of 0.738. Calibration curves for the probability of OS at 1, 3, and 5 years indicated satisfactory consistency between actual observation and nomogram-predicted OS probabilities in both the training cohort and validation cohort (Figure 7B and Supplementary Figure 15). Furthermore, decision curve analysis (DCA) results of the nomograms also confirmed their clinical applicability for predicting the OS, with superior performance compared with AJCC TNM stage. Thus, the results showed that the nomogram expressed a higher net prognostic benefit than the TNM staging system (Figure 7C and Supplementary Figure 15).

### Evaluation of the Expression and Distribution of the Three Key Tumor Stemness-Related Genes and Performance of a Stemness-Related Genetic Model

**FIGURE 8:**
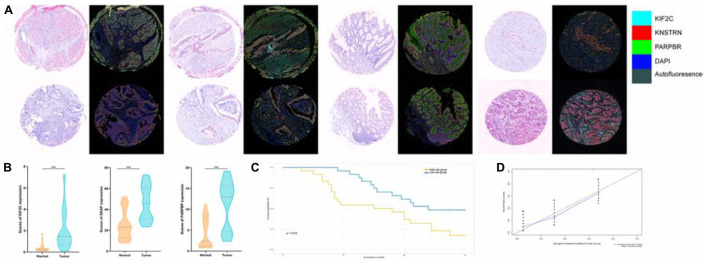
**(A)** Tumor tissue and corresponding non-tumorous adjacent tissue were collected from colorectal cancer patients in TMAs. The expression of PARPBP (green), KNSTRN (red), and KIF2C (cyan) indicates the lipid droplets in tumor-infiltrating myeloid cells. Nuclei (blue) were stained using DAPI. The absolute number of positive cells was quantified in whole fields (hpf; scale bar = 20 lm). **(B)** Comparing the expression of PARPBP, KNSTRN, and KIF2C in tumor tissue and non-tumorous adjacent tissue based on the single index strength score (Student’s two-tailed *t*-tests, ***p < 0.001). **(C)** The Kaplan–Meier test of the risk score for the overall survival between the high-risk and the low-risk group (log-rank test, *p* < 0.05). **(D)** The calibration curves for identifying the consistency between actual observation and nomogram-predicted OS probabilities.

Next, we detected the expression and distribution of PARPBP, KNSTRN, and KIF2C in colorectal cancer and performed a multiplex immunofluorescence staining in TMAs (Figure 8A). We assessed the single index and single index strength score of the three genes in cancer tissue and matched normal tissue samples, which showed that the expression of these genes was higher in cancer tissues based on the single index strength score (Student’s two-tailed *t*-tests, ^∗∗∗^*p* < 0.001, Figure 8B). To further evaluate the performance of the stemness-related genetic model, we calculated the risk scores based on the single index strength score of the three genes and the prognostic index formula (risk scores = [Status of PARPBP ^∗^ (-0.6921)] + [Status of KNSTRN ^∗^ (1.4204)] + [Status of KIF2C ^∗^ -0.9696]). We classified the patients into high- and low-risk groups according to risk score (Table 2), and the Kaplan–Meier OS curves of the two groups were significantly different (log-rank test, *p* < 0.05, Figure 8C). Additionally, we also established calibration curves for the probability of OS at 1, 3, and 5 years, which indicated satisfactory consistency between actual observation and nomogram-predicted OS probabilities in this cohort (Figure 8D and Supplementary Figure 16).

**TABLE 2:** Comparisons of baseline characteristics of CRC patients in low- or high-risk group by risk scores.

**Characteristic**	**Low risk (*n* = 24)**	**High risk (*n* = 24)**	***p*-value**
**Age at RC diagnosis, no. (%), years**			0.057
<60	14 (58.3)	20 (83.3)	
≥60	10 (41.7)	4 (16.7)	
**Sex, no. (%)**			0.562
Female	10 (41.7)	12 (50.0)	
Male	14 (58.3)	12 (50.0)	
**Tumor location, no. (%)**			0.768
Colon	9 (37.5)	10 (41.7)	
Rectal	15 (62.5)	14 (58.3)	
**Stage-T, no. (%)**			0.033
T1	1 (4.1)	0	
T3	16 (66.7)	10 (41.7)	
T4	7 (29.2)	14 (58.3)	
**Stage-N, no. (%)**			0.162
N0	4 (16.7)	4 (16.7)	
N1	12 (50.0)	6 (25.0)	
N2	8 (33.3)	14 (58.3)	
**Stage-M, no. (%)**			0.683
M0	4 (16.7)	3 (12.5)	
M1	20 (83.3)	21 (87.5)	
**Tumor grade, no. (%)**			0.017
Grade III	19 (79.2)	11 (45.8)	
Grade IV	5 (20.8)	13 (54.2)	

*p-value was calculated using χ^2^-test for categorical variables.*

*CRC, colorectal cancer.*

## Discussion

According to the recently developed CSC hypothesis, tumor cells are suggested to originate from a stem cell population with self-renewal capacity (Wicha et al., 2006). These CSCs have been reported to be involved in resistance to cytotoxic conditions, promoting the propagation and recurrence of cancer (Aghaalikhani et al., 2019). Identifying key genes driving the transformation of tumor CSC and underlying biological mechanisms in colorectal cancer may uncover unprecedented therapeutic targets.

Recent studies indicated that cancer CSCs may be a dynamic population continuously influenced by cooperating forces such as microenvironmental, epigenetic, and genetic factors (MacArthur and Lemischka, 2013; Meacham and Morrison, 2013; Lei et al., 2014). The stem cells in normal colonic crypts are continuously and random replaced by other homologous cells, which can provide an advantage for the accumulation of oncogenic mutations through complex stem cell dynamics (Snippert et al., 2014; Song et al., 2014). Myant et al. (2013) demonstrated that oncogenic mutations accumulated in stem cells may trigger the rapid development of aggressive subclones in colon adenomas. The cancer stem cells (CSCs) can compete with normal stem cells, which could be promoted through genetic mutations as well as environmental pressures (including radiotherapy and chemotherapy) (Humphries et al., 2013). Our results revealed a higher proportion of TP53 mutant cells in the high-mRNAsi group than in the low-mRNAsi group. Previous research showed that mutations of TP53 can shut down its tumor suppressor function, promoting the self-renewal, reprogramming, and differentiation of CSCs (Brosh and Rotter, 2009; Emmink et al., 2013).

Analysis of tumor infiltration indicated increased infiltration by CD8^+^ and CD4^+^ T cells in the high-mRNAsi group of colon cancer. Cancer infiltration by CD8^+^ T cells may predict higher sensitivity to immunotherapy and better prognosis. A recent study revealed stem-like CD8^+^ T-cell populations that are able to proliferate and produce high levels of checkpoint molecules under stimulation, with the ability to clonally expand to functional effector T cells and self-renew (Rizvi et al., 2018; Skoulidis et al., 2018; Wu et al., 2019). Tumors with high levels of stemness may have higher levels of infiltration by immune cells such as CD8^+^ and CD4^+^ T cells, as well as having the potential to produce neoantigens that sensitize them to treatment with immune checkpoint inhibitors. Although TME and stemness were both identified as significant features of cancer in recent years, their covariation across cancers has not been systematically investigated. We found that TMB is higher in the high-mRNAsi group than in the low-mRNAsi group of both colon and rectal cancer. The TMB can be defined as the amount of non-synonymous mutations in protein-coding regions, which may promote the production of neoantigens by tumor cells (Pai et al., 2017). Recent studies established the significance of TMB as a predictive biomarker for the success of treatment with immune checkpoint inhibitors (ICIs) such as anti-programmed cell death (PD)-1 and anti-programmed death-ligand 1 (PD-L1) therapy (Rizvi et al., 2015; Rimm et al., 2017; Jansen et al., 2019). In this study, we found that tumors with a high mRNAsi may potentially be more easily recognized by the immune system and, therefore, more sensitive to treatment with immune checkpoint inhibitors.

To further explore the prognostic value and biological mechanisms of potential therapeutic targets, we built a cancer stemness-related prognostic model to provide novel insights into treatment options for colon and rectal cancer. The prognostic index is based on the fractions of three genes identified among the cancer stemness-related key module genes. PARPBP, a significant inhibitor of homologous recombination (HR), has been demonstrated to be related to increased AFP levels, proliferation, differentiation, and poor prognosis of lung and hepatocellular carcinoma patients (Yan et al., 2018; Su et al., 2020). PARPBP promotes tumor cell migration and invasion by enhancing mutagenic replication, extravasation, anoikis resistance, and self-renewal in lung cancer (Xu et al., 2019). KNSTRN, an important component of the mitotic spindle, was found to regulate chromosome segregation and anaphase onset during mitosis in cancer cells (O’Connor et al., 2013). Furthermore, recent studies demonstrated that accumulation of KNSTRN mutations may be an early event in cancer development that accelerates tumor growth in cutaneous squamous cell carcinoma and melanoma (Choi et al., 2016; Schmitz et al., 2019). KIF2C is an important regulator of chromosome segregation, bipolar spindle formation, and microtubule depolymerization during mitosis, and it may be related with poor prognosis in non-small cell lung cancer (Lee et al., 2014). KIF2C was shown to act as a tumor antigen that can elicit spontaneous and frequent CD41 T-cell responses of the Th1 type in colorectal cancer, in a process that is influenced by peripheral T-regulatory cells (Gwon et al., 2012).

Furthermore, we established a nomogram to better predict the survival of colon and rectal cancer patients and visualize the prediction results, which can further improve the compliance and therapeutic efficacy for patients. Additionally, we compared the prognostic accuracy of the TNM stage and nomogram model with DCA, which showed that the nomogram model consisting of stemness-related genes and clinical phenotype could have a higher prognostic ability than the TNM stage. The present results suggest that the model based on three stemness-related genes may have reliable prognostic accuracy in conjunction with the clinical phenotype.

However, some of the data in this retrospective analysis released in publicly available datasets may be limited, and the incomplete clinicopathological information may cause potential bias that would influence the evaluation of the prognostic ability. Data from TCGA are from Western countries, and all of the datasets consisted of a mutational landscape and transcriptome, which may hinder the clinical translation and generalization of the prognostic model. Consequently, we performed multiplex immunofluorescence staining in TMAs of colorectal cancer patients from China and verified the prognostic ability of the nomogram model in an advanced colorectal cancer cohort. As shown in Figure 2, stage IV colon cancer patients with higher mRNAsi values had a lower apparent survival probability than those with lower mRNAsi values, and the difference was statistically significant, which suggested a correlation between CSCs and advanced tumor. The evaluation of CSC-related genes may be a prognostic marker for selecting patients at high risk of metastasis from CRC, who are likely to benefit from treatment. Immunofluorescence results could be employed to evaluate the expression and distribution of the three key stemness-related genes identified by proteomics, with the potential to make this model more convenient and reliable in clinical practice.

## Conclusion

Taken together, our study highlights a robust correlation between the level of cancer stemness and traits related to tumor heterogeneity, including the immune microenvironment, TMB, and the expression of m6A RNA methylation regulatory factors in colorectal cancer cells. The prognostic signature based on mRNAsi may contribute to personalized prognosis of clinical outcomes in colorectal cancer and act as a potential prognostic biomarker for responses to differentiation therapies in clinical practice. The proposed stemness-related genetic model could provide great assistance in formulating efficient therapeutic strategies for the personalized management of colorectal cancer.

## Data Availability Statement

The datasets presented in this study can be found in online repositories. The names of the repository/repositories and accession number(s) can be found in the article/Supplementary Material.

## Ethics Statement

This study was approved by the Institutional Review Board of National Cancer Center (NCC), Chinese Academy of Medical Sciences (approval no. 2017–20). All the patients’ samples used in this study have obtained necessary consent.

## Author Contributions

RW, JQ, ZJ, and XW: conception and design, collection, and assembly of data. RW, JQ, and SL: data analysis and interpretation. All authors: manuscript writing, final approval of the manuscript, and accountable for all aspects of the work.

## Conflict of Interest

The authors declare that the research was conducted in the absence of any commercial or financial relationships that could be construed as a potential conflict of interest.

## Publisher’s Note

All claims expressed in this article are solely those of the authors and do not necessarily represent those of their affiliated organizations, or those of the publisher, the editors and the reviewers. Any product that may be evaluated in this article, or claim that may be made by its manufacturer, is not guaranteed or endorsed by the publisher.

## Footnotes

1
https://tcga-data.nci.nih.gov/tcga/

2
http://asia.ensembl.org/index.html

3
http://cibersort.stanford.edu/

## Abbreviations

CSC: cancer stem cell
TME: tumor microenvironment
MHC: major histocompatibility complex
COAD: colon adenocarcinoma
READ: rectum adenocarcinoma
OCLR: one-class logistic regression
CIBERSORT: Cell type Identification by Estimating Relative Subsets of RNA Transcripts
ESTIMATE: Estimation of Stromal and Immune cells in Malignant Tumors using Expression data
m6A: N6-methyladenosine
TCGA: The Cancer Genome Atlas
SNV: single nucleotide variants
RNA-seq: RNA-sequencing
DEGs: differentially expressed genes
WGCNA: weighted gene co-expression network analysis
TOM: topological overlap matrix
MM: module membership
GS: gene significance
KEGG: Kyoto Encyclopedia of Genes and Genomes
GO: Gene Ontology
TMAs: tissue microarrays
OS: overall survival
ROC: receiver operating characteristic
AUC: area under the curve
lncRNA: long non-coding RNA.
