# Supplementary material for: Integrative Analysis of Biomarkers Through Machine Learning Identifies Stemness Features in Colorectal Cancer
Source: Front Cell Dev Biol. 2021 Sep 8;9:724860. doi: 10.3389/fcell.2021.724860 (PMC8456021; doi:10.3389/fcell.2021.724860)
Supplement: Supplementary file 1 [file Table_1.DOCX]

**Supplementary** Materials

Study design

To explore the mRNAsi in colon and rectal cancer, we obtained the transcriptome profiling for the gene expression and clinical information of colon and rectal cases from the TCGA database, and the analysis workflow was shown (supplement Figure 1). We employed the "Cell type Identification by Estimating Relative Subsets of RNA Transcripts (CIBERSORT)" algorithm and “Estimation of Stromal and Immune cells in Malignant Tumors using Expression data (ESTIMATE)” to develop the immune score model construction and identify the proportions of immune cells with support vector regression. And we have analyzed the alteration spectrum reversible N6-methyladenosine (m6A) RNA methylation regulators in different level of mRNAsi colon and rectal cancer group. Besides we assessed stemness-related genes sets with WGCNA. We identified signatures from a key gene module, and their prognostic role in colon and rectal cancer. Our studies applied some multi-omics approaches to identify the features of cancer stemness and define the roles of stemness-related genes in colorectal cancer.

**m6A RNA methylation regulators**

Twenty-one m6A RNA methylation regulators, including *METTL16, METTL14, METTL3, RBM15, KIAA1429, WTAP, ZC3H13, YTHDC2, YTHDC1, YTHDF2, YTHDF1, HNRNPC, YTHDF3, FTO, HNRNPC, IGF2BP1, HNRNPA2B1, RBMX, ALKBH5, IGF2BP3, IGF2BP2* [1,2], were used in this study. And mRNA expression data of these m6A RNA methylation regulators were compared between high mRNAsi scores and low mRNAsi scores groups. The package “corrplot” R was employed to identify the Pearson correlations between genes. The correlation between the expression of these genes could be evaluated in different groups.

**Protein–Protein Interaction (PPI) Network**

We used Search Tool for the Retrieval of Interacting Genes (STRING) Version 11.0 (<https://string-db.org/>) to establish the PPI network, in order to determine the co-expression relationship between key module genes [3].

**Oncomine and Cbioportal database**

Oncomine (http://www.oncomine.org/) was employed to identify differences in transcriptomic RNA-sequencing data of module genes between tumors and normal tissues in colorectal cancer. The threshold limits were as follows: p-value, 0.05; fold change, all; gene level, 10%; data type, mRNA. We compared the transcriptome level of key module genes in clinical cancer specimens were determined by Student's t test. And Cbioportal (<http://www.cbioportal.org/>) was used to identified the somatic mutations of module genes in colorectal cancer [4,5].

**Multiplex immunofluorescence image analysis**

The Mantra System (PerkinElmer, Waltham, Massachusetts, US) was used to capture the multispectral immunofluorescence images with the fluorescence spectra at 20-nm wavelength intervals from 420 to 720 nm with the same exposure time, which were then composited to establish a single stack image. To capture images of sections without autofluorescence, we extracted the spectrum of autofluorescence of TMAs and each fluorescein from the images of unstained and single-stained sections, which were used to establish the spectral library for multispectral unmixing using inForm image analysis software (PerkinElmer, Waltham, Massachusetts, US). Two independent pathologists analyzed and counted single-positive cells and the expression of the three genes in each tissue of TMAs at 200× magnification in a blinded manner. The nucleated stained cells were quantified and expressed as the number of cells in TMAs. The positive rate of single index and single index intensity score were employed to evaluate the expression and distribution of the identified key genes in colorectal cancer, which were calculated by multiplication of the multiplex immunofluorescence staining intensity (percentage of single index %= Number of positive cells/ Total number of cells; The 25% staining was taken as the threshold of the strength score; 25–49%, strength I; 50–74%, strength II; 75–100%, strength III; single index strength score=[(strength I*positive rate of single index) *1+(strength II*positive rate of single index) *2+(strength III*positive rate of single index) *3] *100) [6].

**Reference**

1. Hänzelmann S, Castelo R, Guinney J. GSVA: gene set variation analysis for microarray and RNA-seq data. BMC bioinformatics. 2013;14:7. Epub 2013/01/18. doi: 10.1186/1471-2105-14-7. PubMed PMID: 23323831; PubMed Central PMCID: PMCPMC3618321.

2. Newman AM, Liu CL, Green MR, Gentles AJ, Feng W, Xu Y, et al. Robust enumeration of cell subsets from tissue expression profiles. Nature methods. 2015;12(5):453-7. Epub 2015/03/31. doi: 10.1038/nmeth.3337. PubMed PMID: 25822800; PubMed Central PMCID: PMCPMC4739640.

3. Langfelder P, Horvath S. WGCNA: an R package for weighted correlation network analysis. BMC bioinformatics. 2008;9:559. Epub 2008/12/31. doi: 10.1186/1471-2105-9-559. PubMed PMID: 19114008; PubMed Central PMCID: PMCPMC2631488.

4. Cerami E, Gao J, Dogrusoz U, Gross BE, Sumer SO, Aksoy BA, Jacobsen A, Byrne CJ, Heuer ML, Larsson E, Antipin Y, Reva B, Goldberg AP, Sander C, Schultz N. The cBio cancer genomics portal: an open platform for exploring multidimensional cancer genomics data. Cancer Discov. 2012 May;2(5):401-4. doi: 10.1158/2159-8290.CD-12-0095. Erratum in: Cancer Discov. 2012 Oct;2(10):960. PMID: 22588877; PMCID: PMC3956037.

5. Gao J, Aksoy BA, Dogrusoz U, Dresdner G, Gross B, Sumer SO, Sun Y, Jacobsen A, Sinha R, Larsson E, Cerami E, Sander C, Schultz N. Integrative analysis of complex cancer genomics and clinical profiles using the cBioPortal. Sci Signal. 2013 Apr 2;6(269):pl1. doi: 10.1126/scisignal.2004088. PMID: 23550210; PMCID: PMC4160307.

6. Szklarczyk D, Gable AL, Lyon D, Junge A, Wyder S, Huerta-Cepas J, et al. STRING v11: protein-protein association networks with increased coverage, supporting functional discovery in genome-wide experimental datasets. Nucleic acids research. 2019;47(D1):D607-d13. Epub 2018/11/27. doi: 10.1093/nar/gky1131. PubMed PMID: 30476243; PubMed Central PMCID: PMCPMC6323986.

**Supplementary Figures**


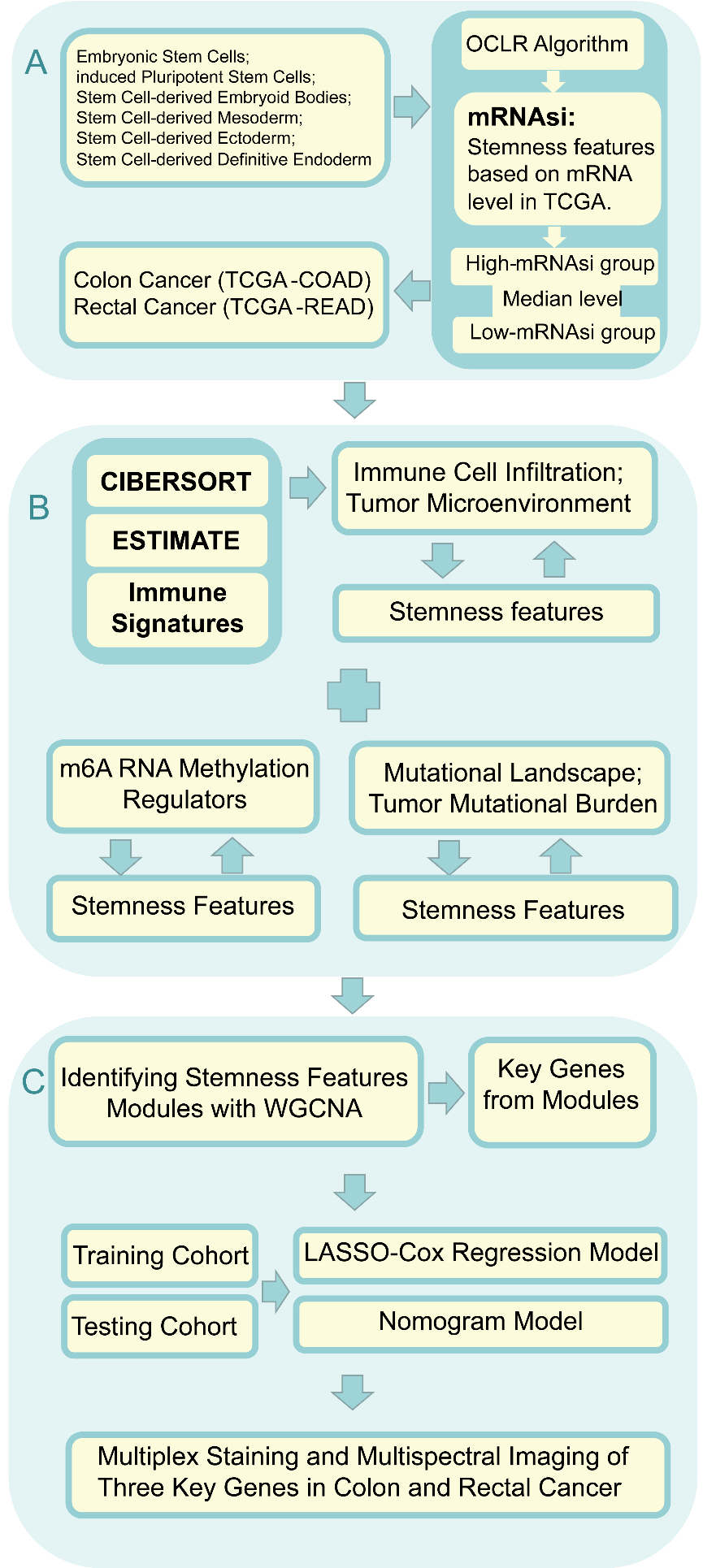


**Supplementary Figure 1.** The analysis process of this study.

**
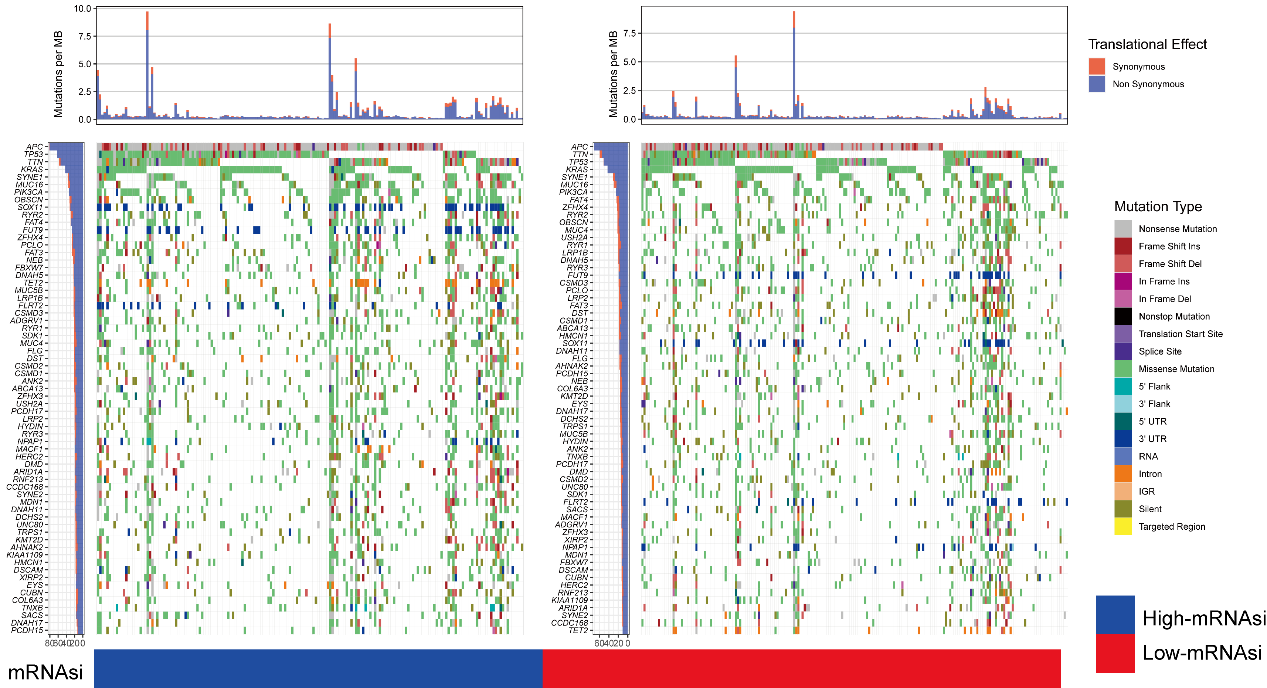
**

**Supplementary Figure 2.** Association of the mRNAsi index with the colon cancer mutational landscape.

**
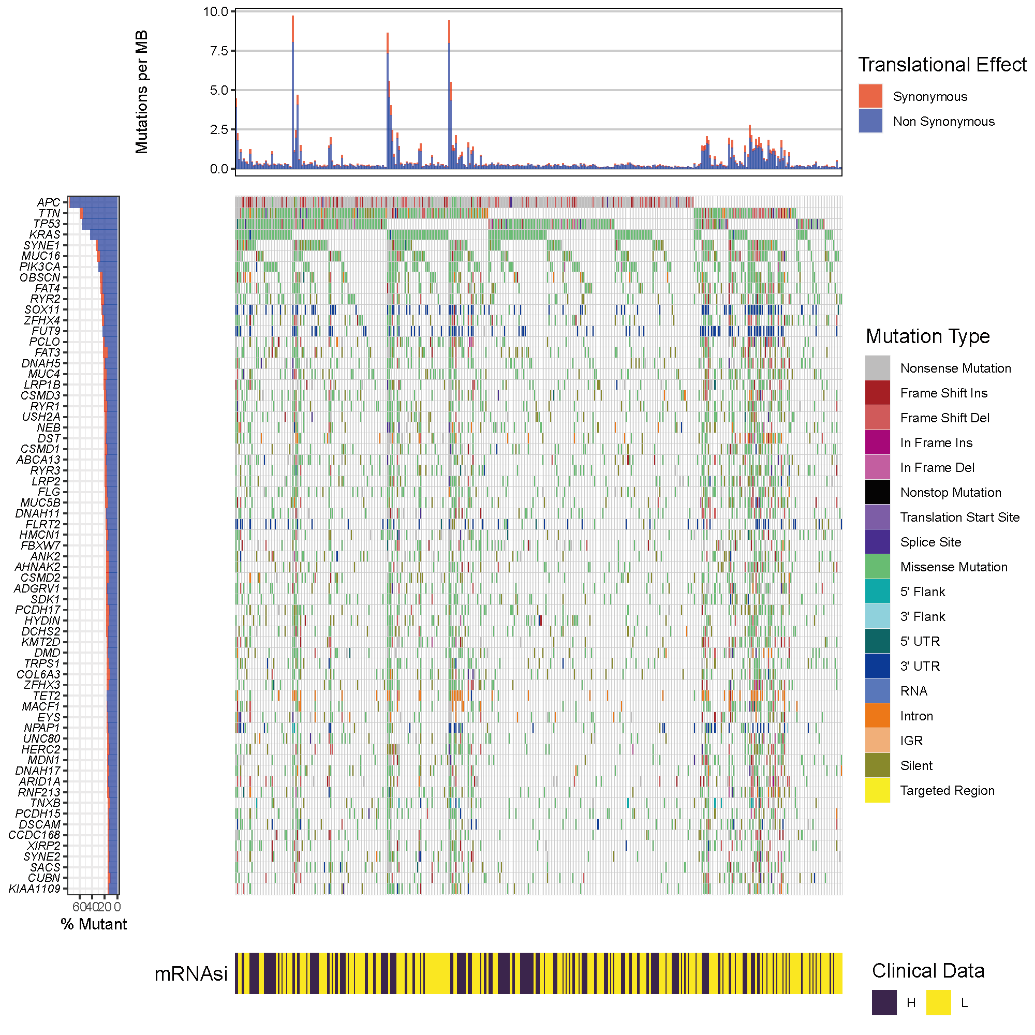
**

**Supplementary Figure 3.** Association of the mRNAsi index with the rectal cancer mutational landscape.

**
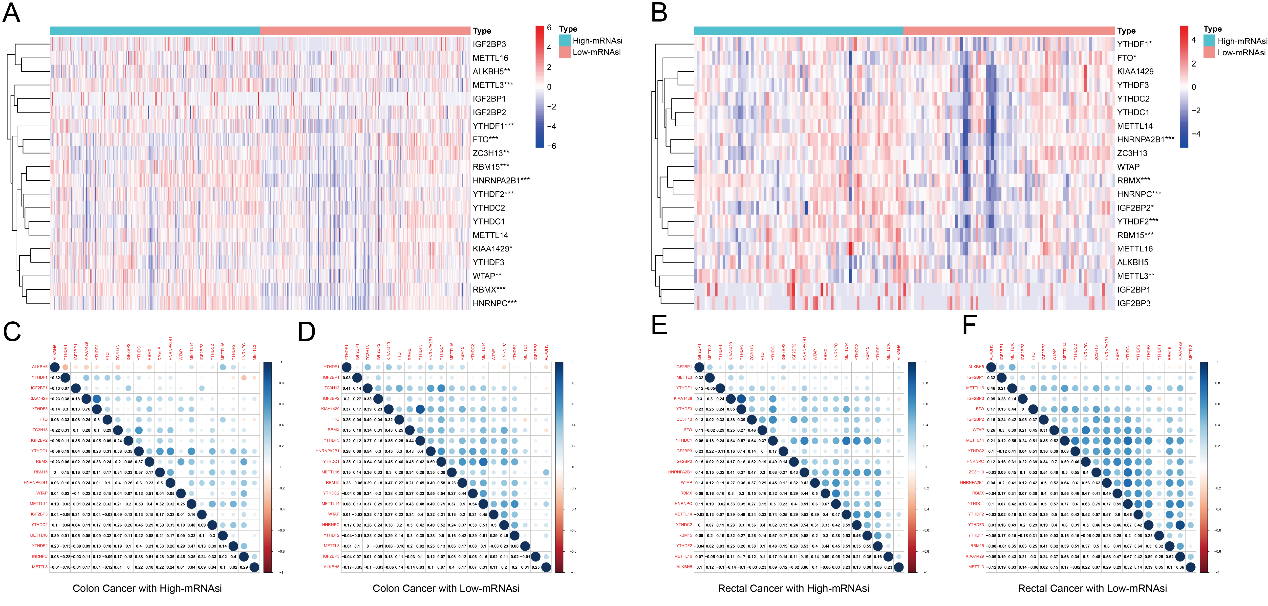
**

**Supplementary Figure 4.** The expression of m6A RNA methylation regulative factors between different level of mRNAsi index. (A, B) The heatmap was used to visualize the expression levels of m6A RNA methylation regulators in different level of mRNAsi index group of colon and rectal cancer. (C-F) The Pearson correlation analysis was used to determine the correlation among m6A RNA methylation regulators.


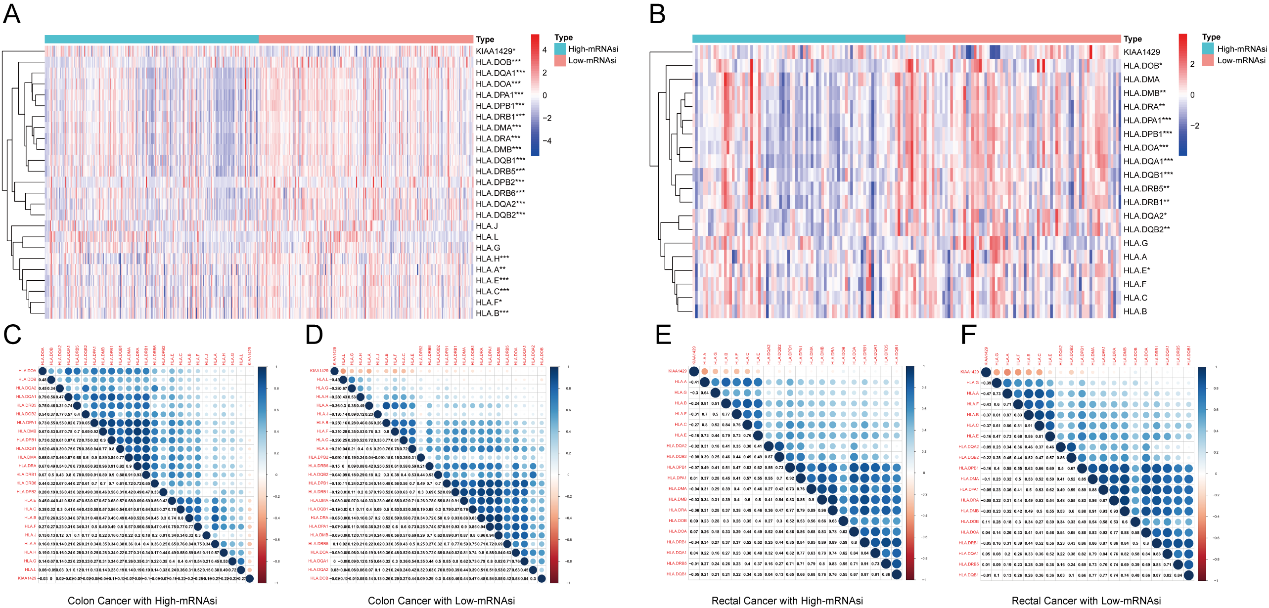


**Supplementary Figure 5.** The expression of HLA between different level of mRNAsi index. (A, B) The heatmap was used to visualize the expression levels of HLA RNA methylation regulators in different level of mRNAsi index group of colon and rectal cancer. (C-F) The Pearson correlation analysis was used to determine the correlation among HLA RNA methylation regulators.

**
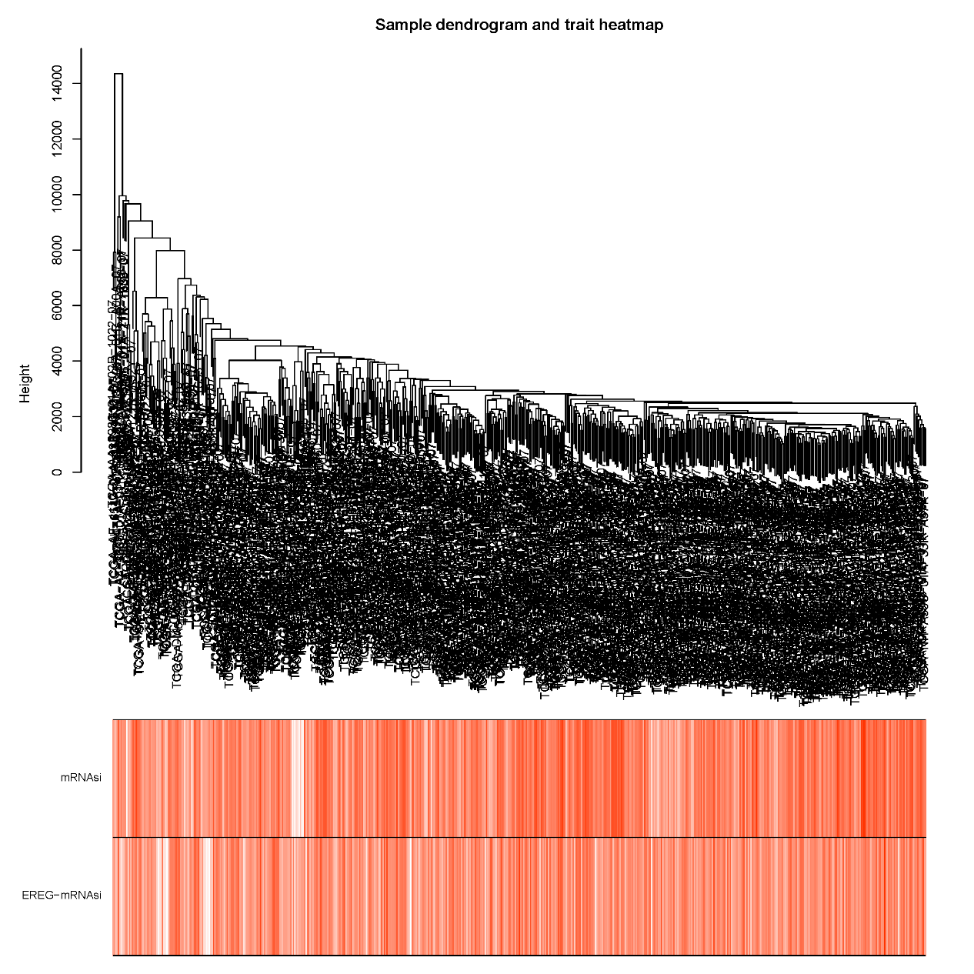
**

**Supplementary Figure 6.** WGCNA for COAD

**
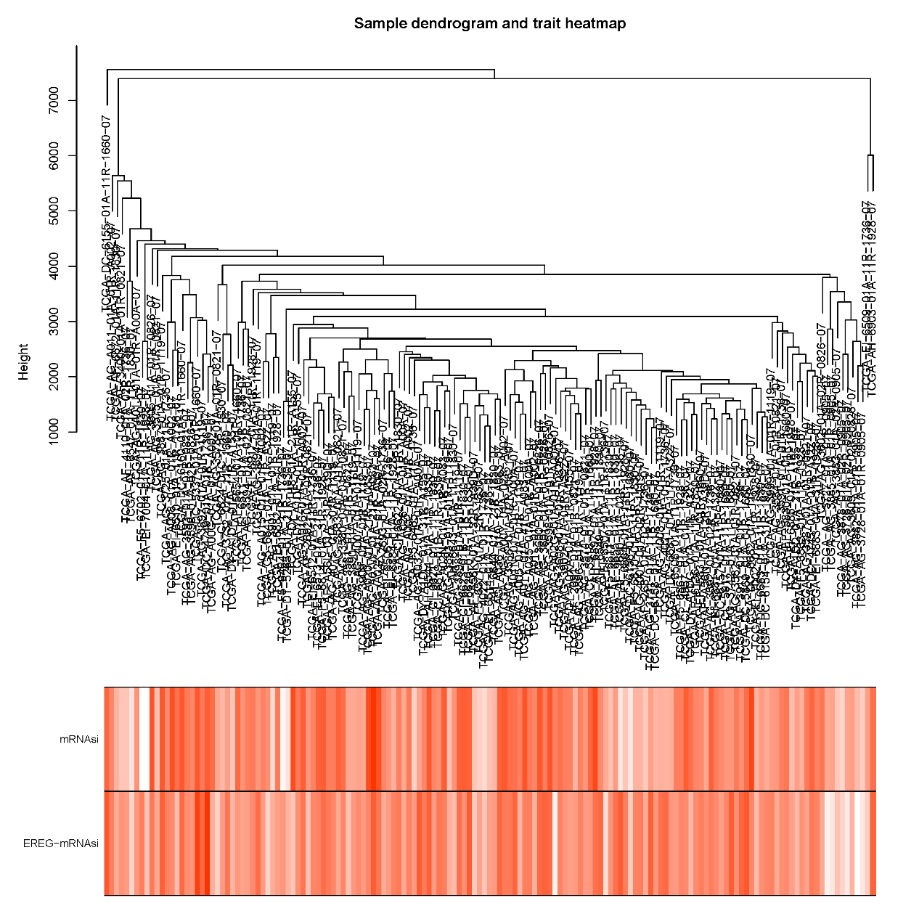
**

**Supplementary Figure 7.** WGCNA for READ

**
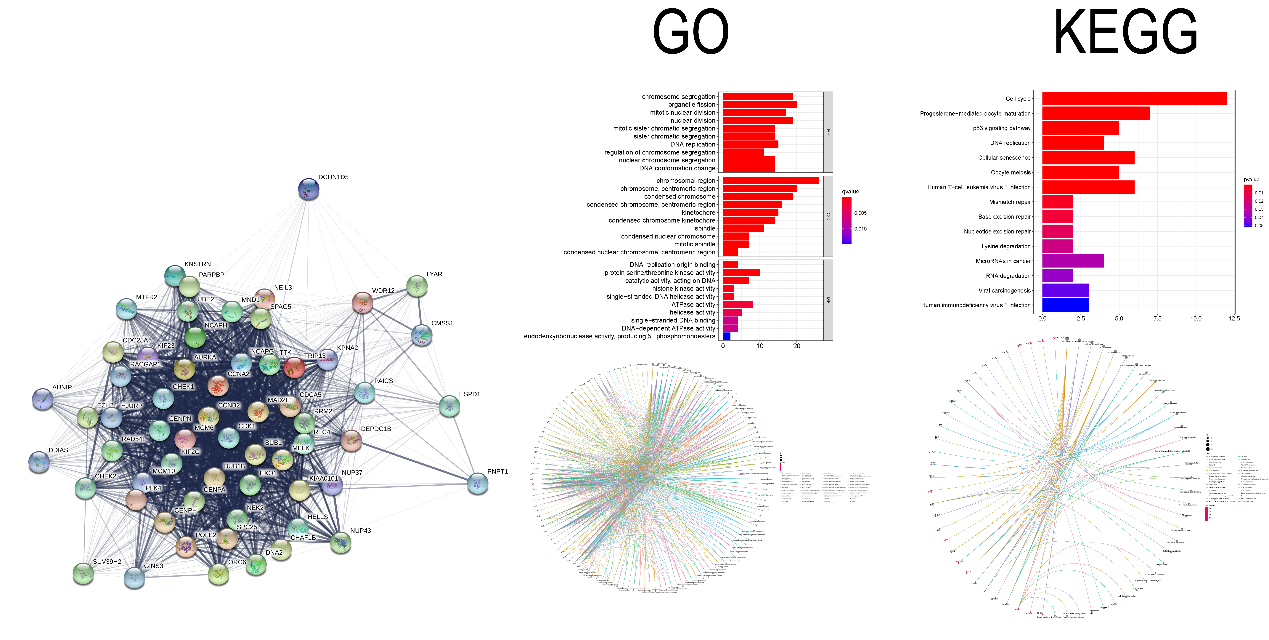
**

**Supplementary Figure 8.** PPI network to, GO and KEGG pathway enrichment analyses for colon cancer.


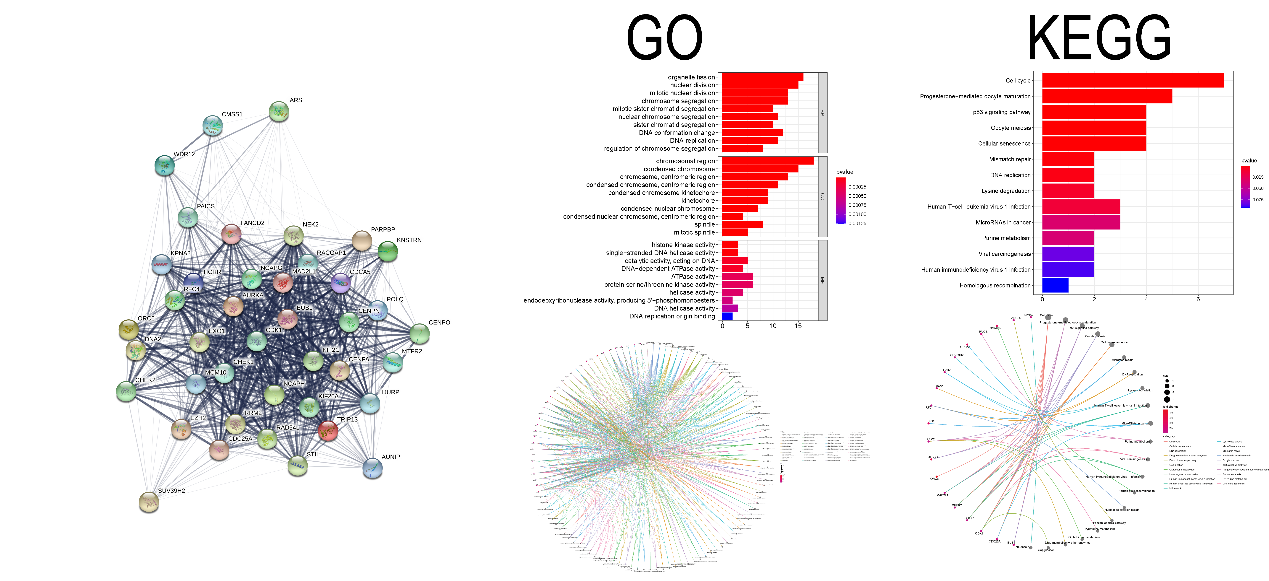


**Supplementary Figure 9.** PPI network to, GO and KEGG pathway enrichment analyses for colon cancer.


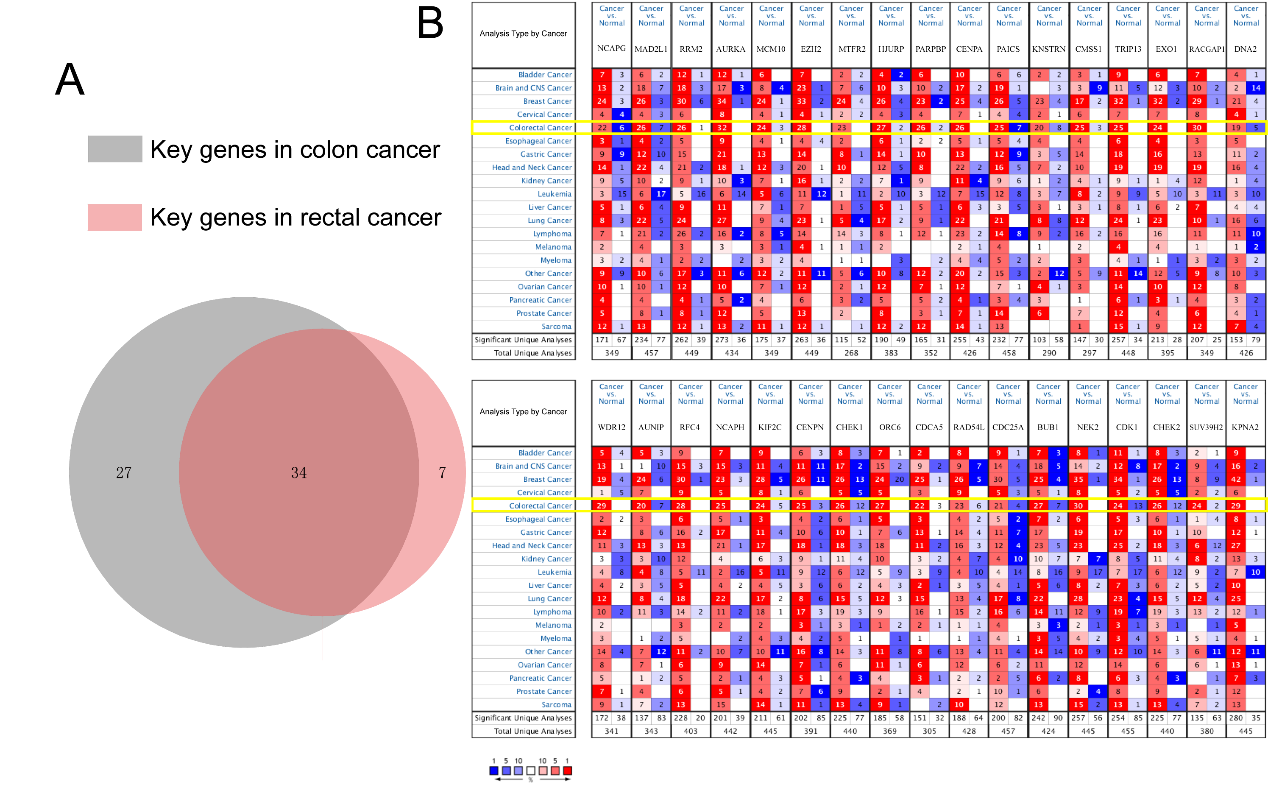


**Supplementary Figure 10.**  Expression validation of key genes. (A) The venn diagram. (B) The mRNA expression of key genes in multiple cancer types from the Oncomine database. The number in the cells represents the number of analyses meeting the thresholds. Red indicates a higher expression level of target genes in tumor tissues than in normal tissues, and blue indicates an opposite expression pattern. The color depth of each cell indicates the gene rank, and the deeper the color depth is, the higher the gene rank.


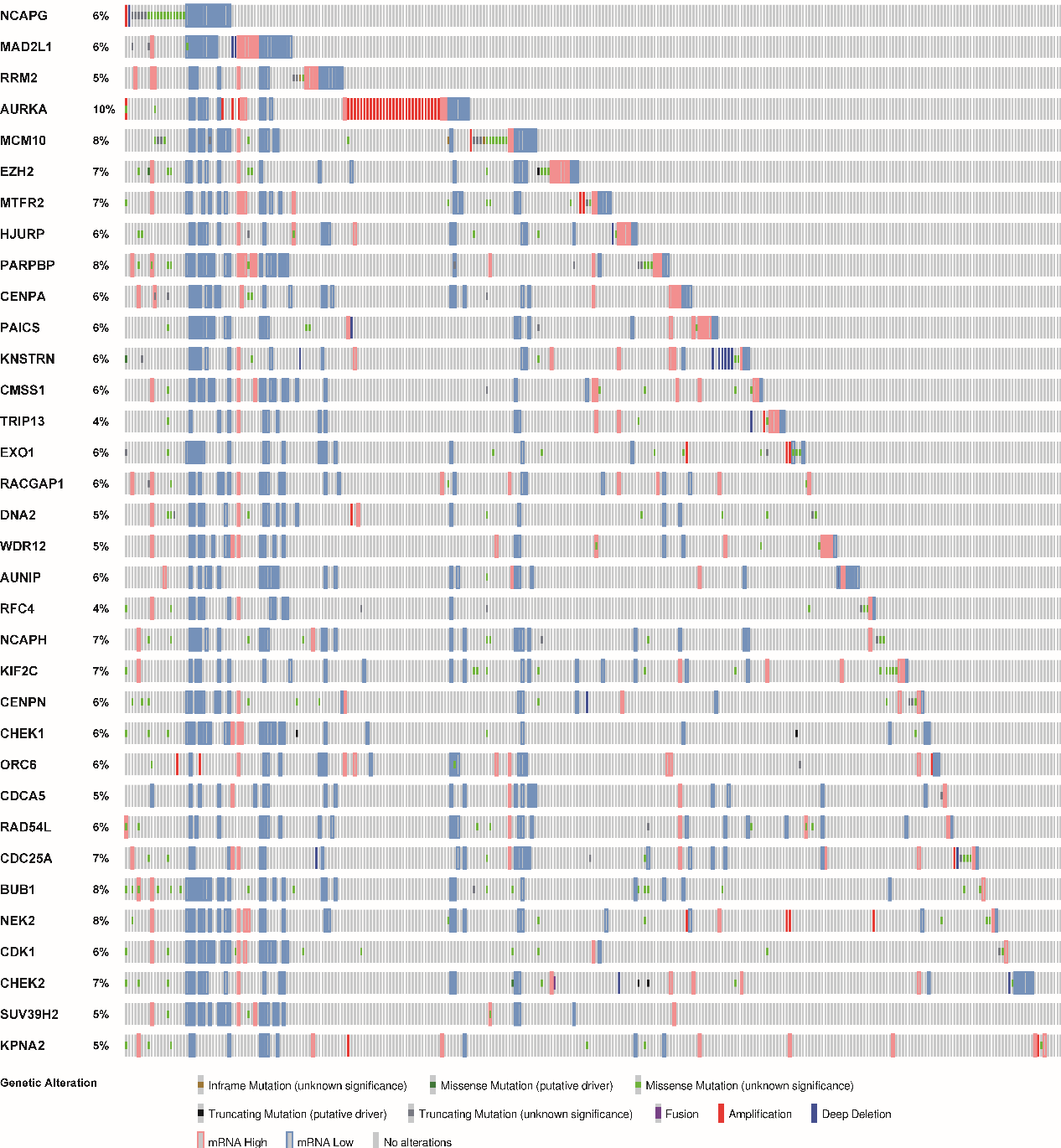


**Supplementary Figure 11.** Mutational landscape of key genes.


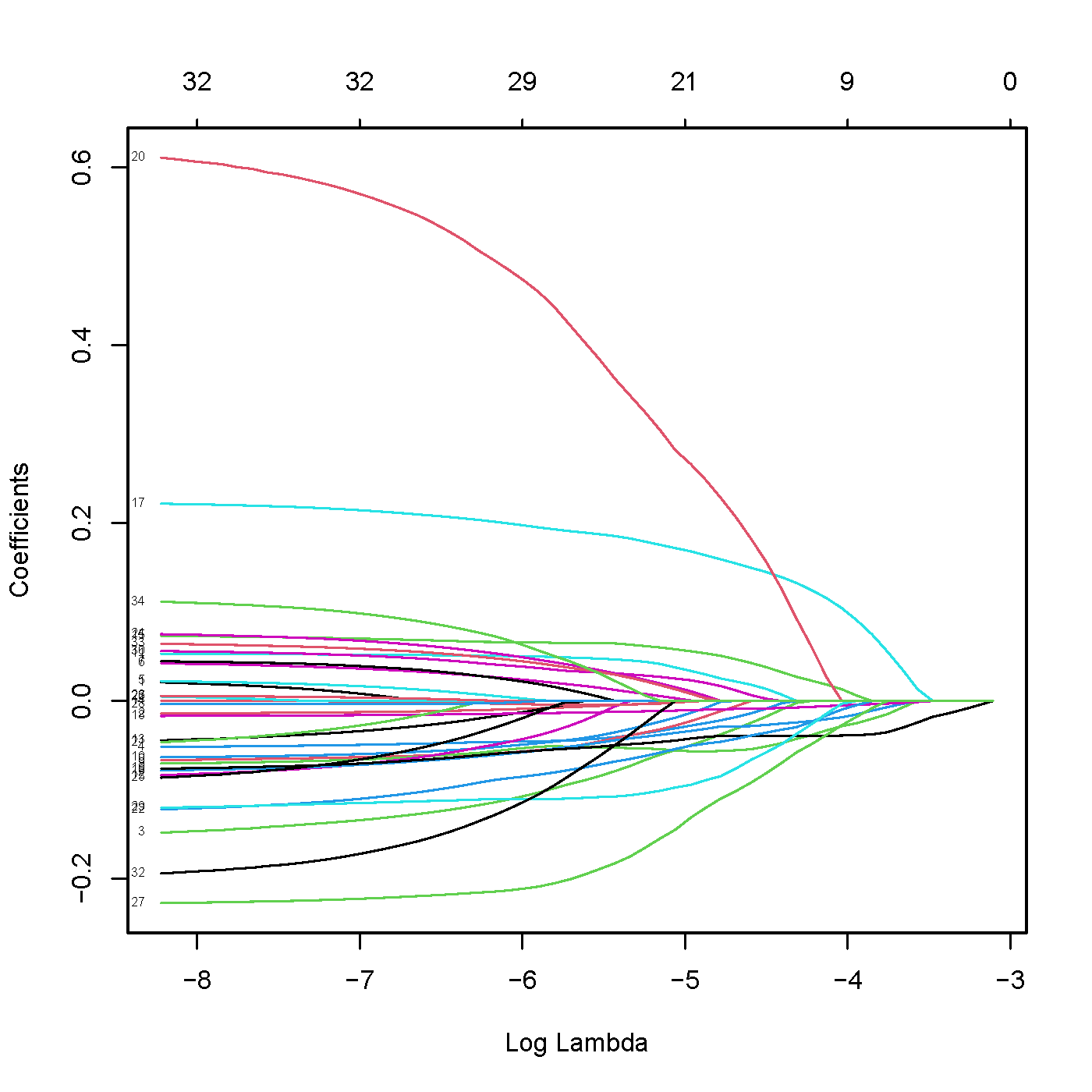


**Supplementary Figure 12.** LASSO algorithm.


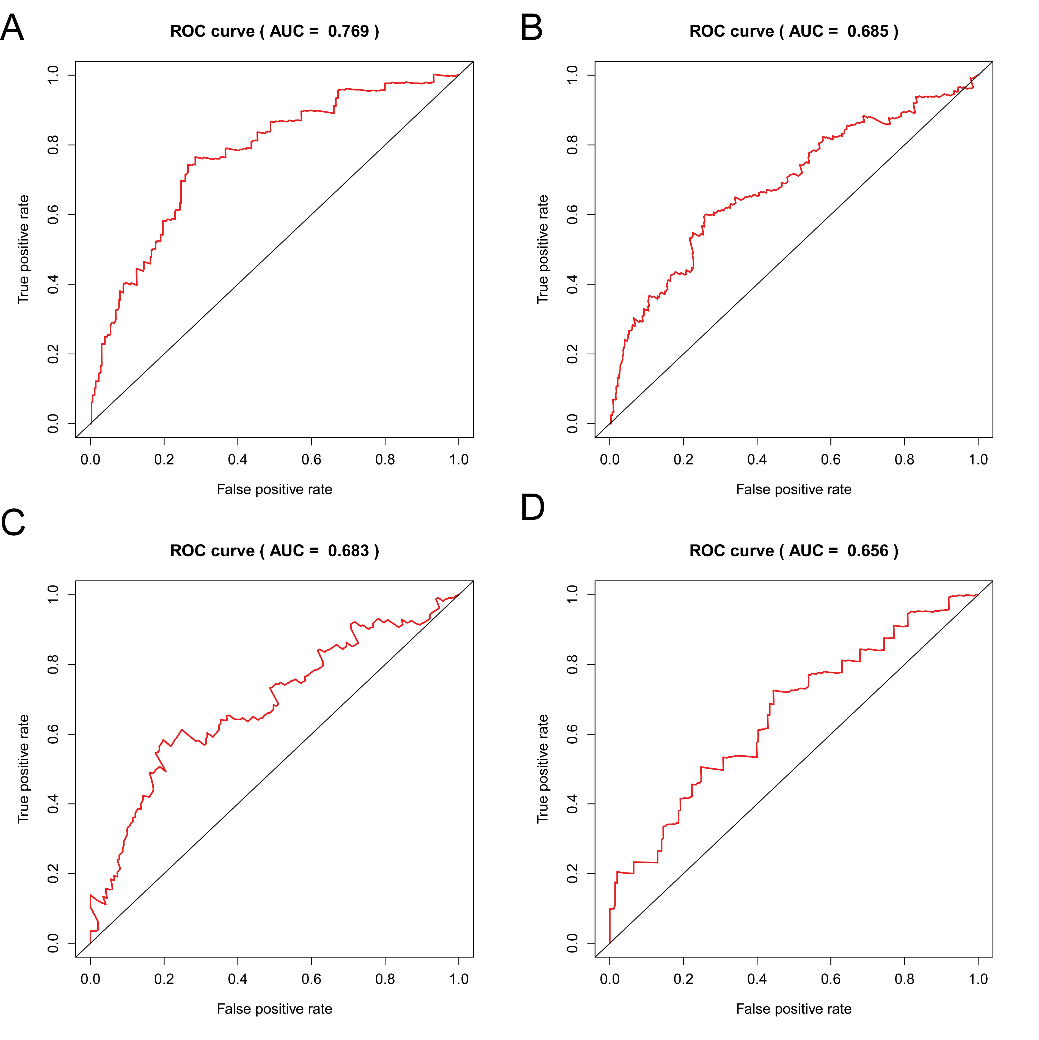


**Supplementary Figure 13.** (A) The prognostic value of the risk score showed by the time-dependent ROC curve for predicting the 3 years overall survival in training group; (B) The prognostic value of the risk score showed by the time-dependent ROC curve for predicting the 3 years overall survival in testing group; (C) The prognostic value of the risk score showed by the time-dependent ROC curve for predicting the 1 years overall survival in training group; (D) The prognostic value of the risk score showed by the time-dependent ROC curve for predicting the 1 years overall survival in testing group;

**
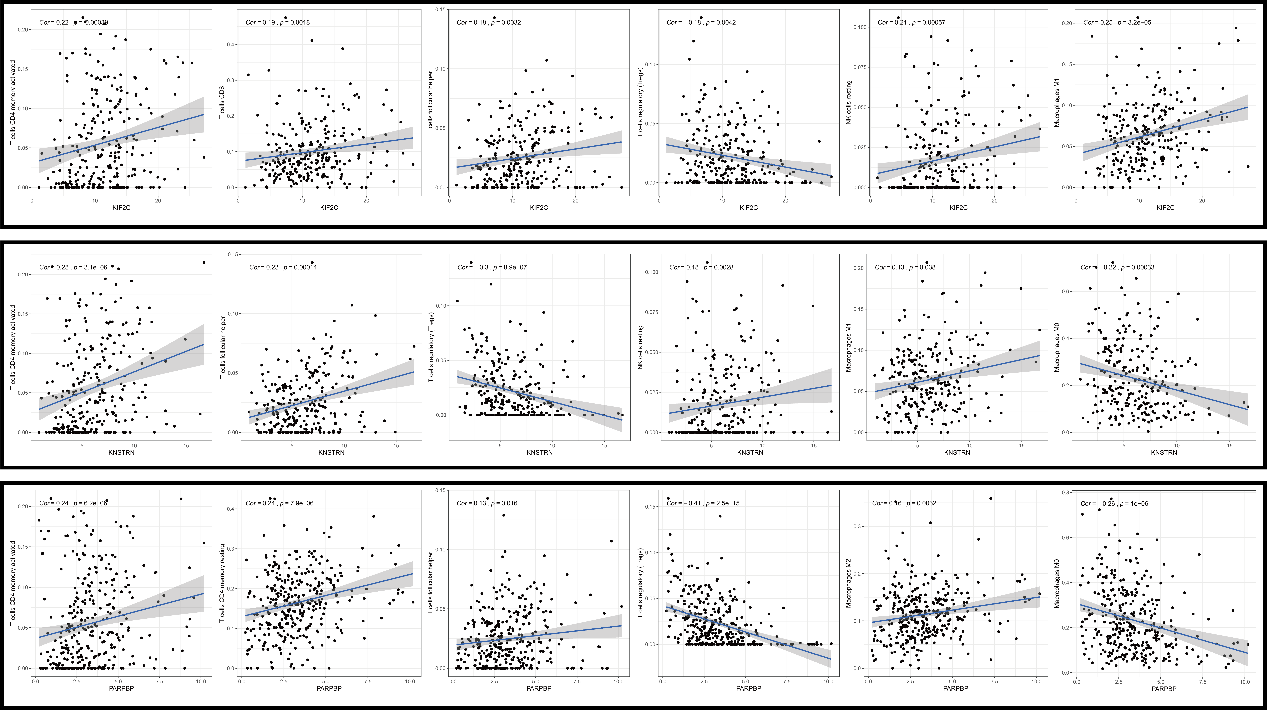
**

**Supplementary Figure 14.** The corrections between the three genes and 22 types of immune cell infiltration profiles.


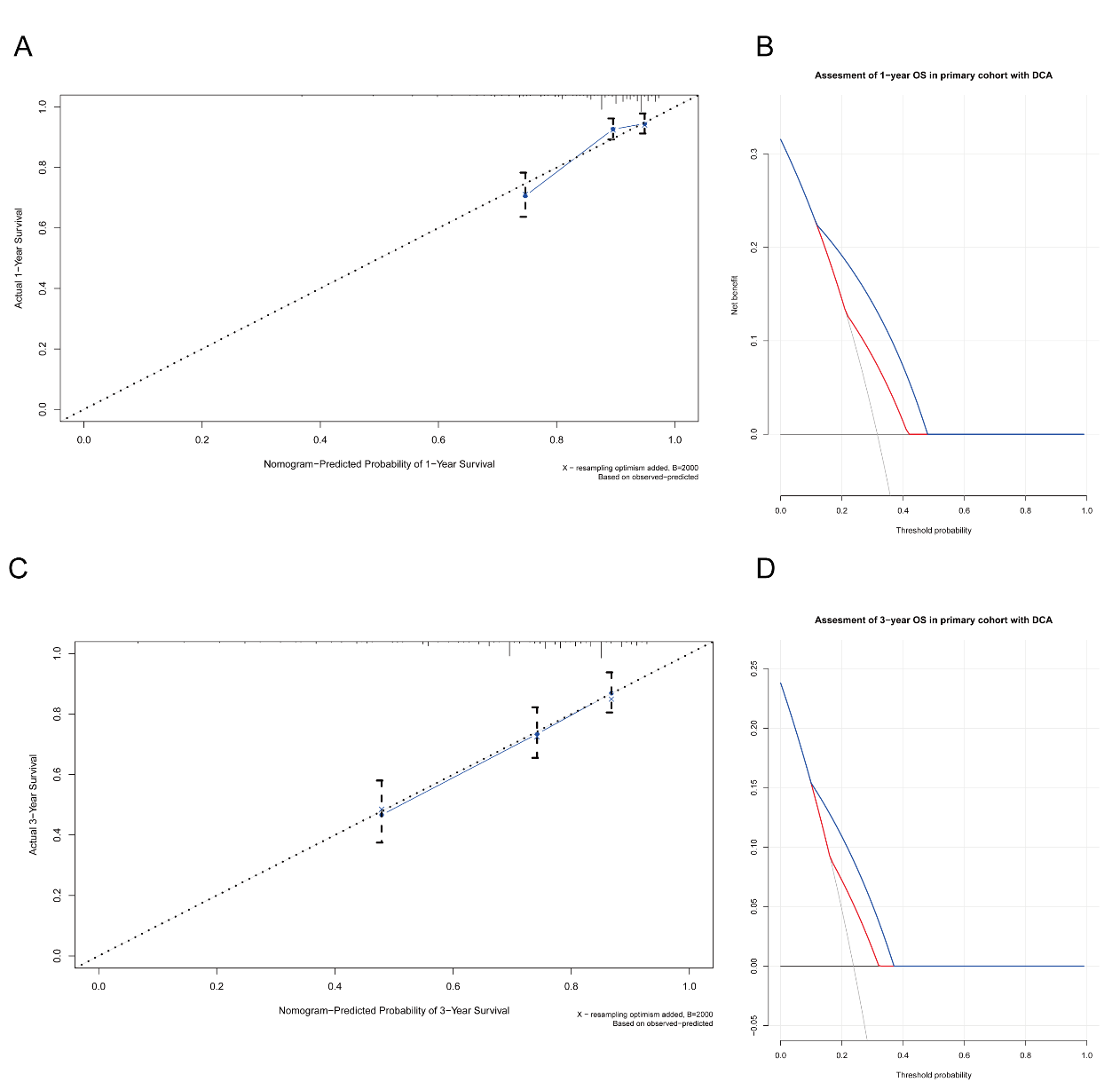


**Supplementary Figure 15.** (A) Calibration curves for the probability of OS at 3 years. The nomogram cohort was divided into three equal groups for validation. The gray line represents the perfect match between the actual (y-axis) and nomogram-predicted (x-axis) survival probabilities. Black circles represent nomogram-predicted probabilities for each group, and X’s represent the bootstrap-corrected estimates. Error bars represent the 95% CIs of these estimates. A closer distance between two curves suggests higher accuracy. (B) The DCA of nomogram in training set for 3 years OS. (C) Calibration curves for the probability of OS at 1 years. The nomogram cohort was divided into three equal groups for validation. The gray line represents the perfect match between the actual (y-axis) and nomogram-predicted (x-axis) survival probabilities. (D) The DCA of nomogram in training set for 1 years OS.


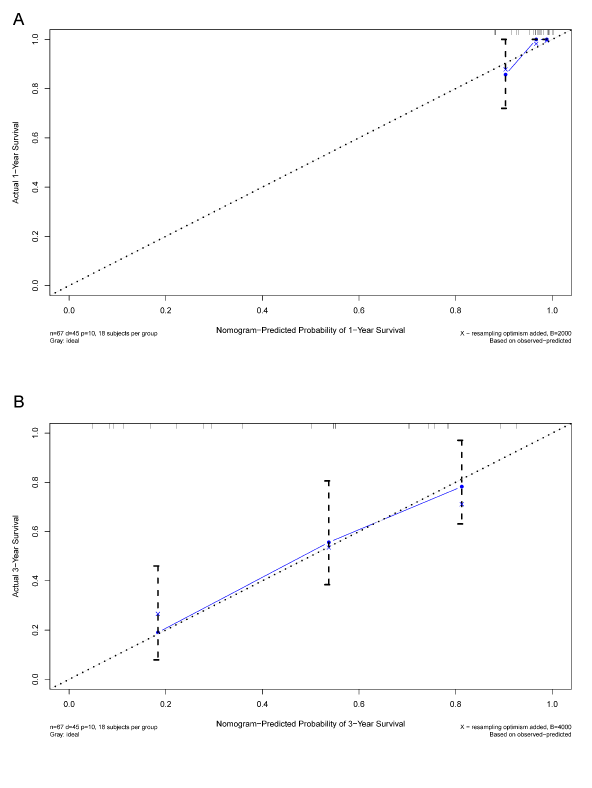


**Supplementary Figure 16.** (A) Calibration curves for the probability of OS at 3 years. The nomogram cohort was divided into three equal groups for validation. The gray line represents the perfect match between the actual (y-axis) and nomogram-predicted (x-axis) survival probabilities. Black circles represent nomogram-predicted probabilities for each group, and X’s represent the bootstrap-corrected estimates. Error bars represent the 95% CIs of these estimates. A closer distance between two curves suggests higher accuracy. (B) Calibration curves for the probability of OS at 1 years. The nomogram cohort was divided into three equal groups for validation. The gray line represents the perfect match between the actual (y-axis) and nomogram-predicted (x-axis) survival probabilities.
